# PLGA-Encapsulated Pterostilbene Mitigates Cadmium-Induced Nephrotoxicity in Rats by Modulating Oxidative Stress, Inflammation, and Apoptosis

**DOI:** 10.3390/toxics14100856

**Published:** 2026-09-26

**Authors:** Fahd Alaajam, Ekramy M. Elmorsy, Amro Duhduh, Abdulrahman S. Aldaghmi, Fahad Alshammari, Ola A. Habotta, Manal S. Fawzy, Shaimaa A. Shehata

**Affiliations:** 1 Department of Medical Laboratory Technology, College of Nursing and Health Science, Jazan University, Jazan 45142, Saudi Arabia; falaajam@jazanu.edu.sa (F.A.); aduhduh@jazanu.edu.sa (A.D.); 2 Center for Health Research, Northern Border University, Arar 73213, Saudi Arabia; ekramy.elmorsy@nbu.edu.sa; 3 Department of Biology, College of Science, Jouf University, Sakaka 72341, Saudi Arabia; asaldaghmi@ju.edu.sa (A.S.A.); fahadm@ju.edu.sa (F.A.); 4 Department of Forensic Medicine and Toxicology, Faculty of Veterinary Medicine, Mansoura University, Mansoura 35516, Egypt; ola_ali@mans.edu.eg; 5 Horus Research Center, Horus University—Egypt (HUE), New Damietta 34518, Egypt; 6 Department of Forensic Medicine and Clinical Toxicology, Faculty of Medicine, Suez Canal University, Ismailia 41522, Egypt; shaimaa_shehata@med.suez.edu.eg

**Keywords:** cadmium nephrotoxicity, mining-cadmium exposure, pterostilbene, PLGA nanoparticles, renal injury, oxidative stress, inflammatory markers, apoptosis-related markers, rat model

## Abstract

Cadmium (Cd) is an environmentally pervasive toxic metal that accumulates in the kidney and promotes oxidative stress, inflammation, and apoptosis. This study evaluated the renoprotective effects of free pterostilbene (PTS) and PLGA-encapsulated PTS (PTS-PLGA) in male Sprague–Dawley rats with Cd-induced nephrotoxicity. Rats received CdCl_2_ (2 mg/kg/day, intraperitoneally) for 3 weeks and oral free PTS or PTS-PLGA at a nominal PTS-equivalent dose of 50 mg/kg/day. The formulation dose was calculated from encapsulation efficiency; however, systemic and renal PTS exposure were not measured. Renal function, kidney injury molecule-1 (Kim-1), oxidative stress, inflammatory, and apoptosis-related markers were assessed together with histopathological, ultrastructural, and immunohistochemical changes. Cd exposure impaired renal function and increased Kim-1, reactive oxygen species, malondialdehyde, protein carbonyls, 8-hydroxy-2′-deoxyguanosine, inflammatory mediators, caspase activities, Bax/Bcl-2 ratio, and p53 expression. These changes were accompanied by reduced antioxidant defenses and Nrf2/HO-1-related markers, as well as marked histological and ultrastructural injury. Both PTS formulations attenuated Cd-associated renal alterations. Under the present dosing conditions, PTS-PLGA was associated with greater improvement than free PTS across most endpoints. PLGA-encapsulated PTS therefore warrants further preclinical investigation as a potential approach to mitigate Cd-induced nephrotoxicity; pharmacokinetic, biodistribution, and empty-carrier control studies are needed to define the basis of the observed formulation-related differences.

## 1. Introduction

Heavy metals are naturally occurring elements with high density and atomic weight that exhibit distinctive physicochemical properties; although certain metals are required at trace concentrations, excessive accumulation may cause toxicity [1,2]. Cadmium (Cd) is a non-essential toxic metal and an important environmental and occupational pollutant [3]. Exposure may occur through mining and smelting activities, battery manufacture, pigments, plastics, contaminated food or water, and tobacco smoke [4,5,6]. The widespread environmental occurrence and persistence of Cd make it a continuing public-health and environmental-toxicology concern. The expanding use of Cd-containing nanomaterials may further increase exposure opportunities in humans and animals [7].

Cadmium exposure is a relevant public-health concern because chronic environmental and occupational exposure has been associated with reduced glomerular filtration and an increased risk of chronic kidney disease (CKD) [8]. Global burden modeling estimated that dietary Cd exposure may account for a median of 12,224 new stage 4–5 CKD cases annually, 2064 deaths, and 70,513 disability-adjusted life years worldwide [9]. Epidemiological evidence further indicates that renal effects may occur at exposure levels below conventional toxicity thresholds; in one analysis, a Cd intake of 23.2 μg/day was associated with a 73% higher CKD risk [10]. Historical and occupational evidence also supports the clinical relevance of Cd nephropathy: environmental Cd exposure was associated with tubular dysfunction, CKD, and osteomalacia in the Japanese itai-itai disease outbreak, while occupational studies have documented increased renal injury among exposed workers [11,12]. In Egypt, an earlier study of Cd-exposed smelting workers reported a 30% prevalence of Cd nephropathy based on blood Cd and urinary β2-microglobulin criteria; this regional observation should be interpreted in the context of its specific occupational setting and diagnostic thresholds [13].

Following inhalation or ingestion, Cd enters the circulation and distributes to multiple organs, with the liver and kidney serving as major long-term depots [4]. The kidney is a principal target of Cd toxicity, particularly because Cd accumulates in proximal tubular epithelial cells. This accumulation can impair mitochondrial integrity and tubular transport, promote cell death, and contribute to proteinuria and acute or chronic kidney injury [2,14]. Thus, experimental models of Cd-induced renal injury are relevant for investigating the health consequences and biomolecular correlates of toxic-metal exposure.

The harmful effects of Cd are largely mediated by excessive generation of reactive oxygen species (ROS), including hydroxyl and superoxide radicals, which induce oxidative stress and damage nucleic acids, lipids, and proteins [4,14,15]. At the same time, Cd depletes endogenous antioxidant defenses, including reduced glutathione (GSH), thereby weakening redox homeostasis [16,17]. Cd-associated oxidative injury is also linked to inflammatory responses and cell-death pathways, which can collectively aggravate renal structural and functional damage [14,18]. The Nrf2/HO-1 axis is an important stress-responsive antioxidant system, whereas TLR4/NF-κB-related signaling contributes to the regulation of inflammatory mediators [19,20]. Persistent oxidative and inflammatory injury may also be accompanied by apoptosis-related changes, including altered Bax/Bcl-2 balance, p53 expression, and caspase activation [21]. Given the widespread environmental presence of Cd and its strong nephrotoxic potential, there is a clear need for renoprotective strategies that attenuate oxidative stress, inflammation, and cell death. Plant-derived natural products are attractive candidates in this context due to their reported antioxidant and anti-inflammatory activities, including in renal disorders [4,22].

Pterostilbene (PTS), a natural dimethylated analog of resveratrol, exhibits antioxidant and anti-inflammatory properties and has shown cytoprotective and renoprotective effects in experimental models [23,24]. PTS is a polyphenolic stilbene rather than a flavonoid. Its physicochemical features, including moderate lipophilicity and low molecular weight, may favor membrane permeability [25,26]. However, its low aqueous solubility, extensive phase II metabolism, and susceptibility to environmental degradation may limit formulation performance and therapeutic application [26]. These properties provide a rationale for evaluating delivery systems designed to improve PTS dispersion and release characteristics.

Polymeric nanoparticles based on biodegradable polymers such as poly(lactic-co-glycolic acid) (PLGA) have been widely investigated as carriers for bioactive compounds, including phytochemicals [27]. PLGA-based formulations can modify the physicochemical properties and in vitro release profile of encapsulated compounds. However, formulation-specific pharmacokinetics, biodistribution, kidney accumulation, and carrier-related biological effects must be directly assessed and were not evaluated in the present study [27,28,29]. Accordingly, the present work evaluates the biological effects of the tested PTS-PLGA formulation rather than claiming kidney-targeted delivery or enhanced systemic bioavailability.

In light of these considerations, this study aimed to evaluate PLGA-encapsulated PTS (PTS-PLGA) in a rat model of Cd-induced nephrotoxicity. We compared free PTS and PTS-PLGA administered at the same nominal PTS-equivalent dose and assessed renal function and injury markers, oxidative-stress and antioxidant indices, Nrf2/HO-1-related markers, TLR4/NF-κB-related inflammatory markers, apoptosis-related markers, and renal histological, ultrastructural, and immunohistochemical changes. We hypothesized that PTS-PLGA would attenuate Cd-associated renal injury and would be associated with more favorable changes in oxidative stress, inflammatory, and apoptosis-related markers than free PTS.

## 2. Materials and Methods

### 2.1. Preparation of Pterostilbene-Loaded PLGA Nanoparticles (PTS-PLGA)

PTS-loaded PLGA nanoparticles were produced by an oil-in-water solvent-evaporation procedure adapted from Kola et al. [30]. Pterostilbene (100 mg) and PLGA (200 mg; 50:50 lactide: glycolide ratio) were first dissolved in 3 mL acetone. This organic solution was introduced gradually into 20 mL of an aqueous poly(vinyl alcohol) solution (1–2%, *w*/*v*) maintained in an ice bath. The mixture was homogenized at 15,000 rpm for 5 min using an Ultra-Turrax T25 homogenizer (IKA-Werke GmbH & Co. KG, Staufen, Germany). The resulting dispersion was subsequently sonicated using a SONOPULS HD 3200 probe sonicator (BANDELIN electronic GmbH & Co. KG, Berlin, Germany) at 80% amplitude, applying 10 s intermittent pulses for the optimized sonication period. The suspension was stirred at room temperature for 3 h using a C-MAG HS 7 magnetic stirrer (IKA-Werke GmbH & Co. KG, Staufen, Germany) to permit acetone evaporation. The resulting PTS-PLGA nanosuspension was kept at 4 °C until subsequent characterization and animal administration.

Particle hydrodynamic diameter, polydispersity index, and zeta potential were determined in triplicate by dynamic light scattering using a Zetasizer Nano ZS (Malvern Instruments Ltd., Malvern, UK) after appropriate sample dilution. The reported mean particle diameter corresponds to the Z-average. Nanoparticle morphology was assessed by transmission electron microscopy (TEM) using a JEM-2100 (JEOL Ltd., Tokyo, Japan) operated at 160 kV. Entrapment efficiency was calculated from the difference between the initial amount of PTS and the amount of unencapsulated PTS, according to the following equation:

EE% = [(Amount of drug initially used − unencapsulated drug)/Amount of drug initially used] × 100.

### 2.2. Fourier-Transform Infrared (FTIR) Analysis

FTIR spectra of free PTS, blank PLGA, and PTS-PLGA nanoparticles were acquired using an FTIR spectrometer (PerkinElmer, Waltham, MA, USA) in transmission mode. Samples were prepared as KBr pellets, and spectra were recorded over 4000–400 cm^−1^. Spectra were qualitatively compared to identify characteristic absorption bands of PTS and PLGA and to assess the spectral profile of the PTS-PLGA formulation.

### 2.3. In Vitro Drug Release

PTS release from the PTS-PLGA nanosuspension was evaluated using a dialysis-based diffusion method in an aqueous release medium. A defined volume of nanosuspension was sealed in a dialysis membrane and immersed in phosphate-buffered saline (PBS; pH 7.4) at 37 °C. At predetermined intervals, aliquots of the external medium were withdrawn for spectrophotometric quantification of released PTS. An equal volume of fresh, prewarmed PBS was added after each withdrawal to maintain the release volume. Cumulative PTS release was calculated as a percentage of the initial PTS amount and plotted against time.

### 2.4. Experimental Animals and Study Design

The in vivo experiment used 60 male Sprague–Dawley rats weighing 240–270 g at study initiation. Animals were supplied by the Medical Experimental Research Center, Mansoura University, Egypt. They were maintained for 2 weeks before treatment in a controlled animal facility at 25 °C and 45% relative humidity under a 12 h light/12 h dark photoperiod, with unrestricted access to standard laboratory chow and water.

The study protocol was reviewed and approved by the Animal Care and Use Committee of the Faculty of Veterinary Medicine, Mansoura University, Egypt (approval no. MU-ACUC; VM.R.26.04.281). All animal procedures complied with the CIOMS/ICLAS International Guiding Principles for Biomedical Research Involving Animals and applicable institutional requirements. The study is reported in accordance with ARRIVE 2.0 guidance.

No prospective sample-size calculation was undertaken. The total sample and group allocation were selected on the basis of the planned experimental design and experience from comparable Cd-induced renal-injury models [31,32,33]. After the acclimatization period, animals were assigned by computer-generated simple randomization to one of six experimental groups, with 10 rats per group:

1. Control: vehicle only.
2. PTS: PTS 50 mg/kg/day by oral gavage [34].
3. PTS-PLGA: PTS-loaded PLGA nanoparticles, equivalent to 50 mg/kg/day PTS.
4. Cd: CdCl_2_ 2 mg/kg/day, i.p. for 3 weeks.
5. Cd + PTS: CdCl_2_ plus PTS 50 mg/kg/day.
6. Cd + PTS-PLGA: CdCl_2_ plus PTS-PLGA (50 mg/kg/day PTS equivalent).

CdCl_2_ (2 mg/kg/day, i.p., 3 weeks) was used to induce chronic nephrotoxicity, as previously described [35]. PTS or PTS-PLGA was administered orally once daily for 3 weeks, 1 h before CdCl_2_ injection. This interval was selected to standardize the repeated-exposure protocol and allow initial oral absorption before each Cd challenge; it was intended to evaluate prophylactic renoprotective potential during ongoing exposure rather than to simulate a clinically established dosing schedule. PTS-PLGA was administered orally at a formulation amount calculated to provide a nominal PTS-equivalent dose of 50 mg/kg/day, based on the formulation entrapment efficiency. The formulation was suspended in distilled water immediately before administration. Because formulation-specific drug loading, pharmacokinetics, and tissue distribution were not determined, dose matching refers to the nominal PTS-equivalent dose rather than confirmed systemic PTS exposure. No animals were excluded during the experiment, and no data points were excluded from the analyses.

### 2.5. Animal Monitoring, Euthanasia, and Tissue Collection

Throughout the treatment period, rats were observed daily for general condition, spontaneous activity, behavior, food and water consumption, and clinical signs of distress. No unexpected adverse events occurred, and no prespecified humane endpoints were reached.

At completion of the 3-week exposure period, animals were food-deprived for 10 h before terminal sample collection. Anesthesia was induced with isoflurane and maintained until the absence of a response to stimulation. Euthanasia was then performed using 5% isoflurane in oxygen; the absence of cardiac and respiratory activity verified death.

Before sample collection, animals within each group were prespecified for one of two analysis streams: seven rats per group were allocated to serum biochemical, renal biochemical, ELISA, fluorescence-based oxidative-stress, caspase, and qRT-PCR analyses; the remaining three rats per group were allocated to histopathological, immunohistochemical, and ultrastructural evaluation. The individual animal was the experimental unit for all statistical comparisons. This allocation was made before tissue harvesting to ensure that fresh or frozen tissues were available for biochemical and molecular analyses, while separately collected tissues could be processed for formalin fixation, paraffin embedding, or TEM resin embedding. Blood was obtained from the retro-orbital venous plexus, permitted to clot, and centrifuged at 3000× *g* for 10 min. The resulting serum was kept at −80 °C until analysis.

Both kidneys were removed promptly, washed in ice-cold 1.15% potassium chloride solution, and apportioned according to the planned downstream analyses. Tissue intended for biochemical and molecular measurements was processed as described below, while samples for light microscopy were fixed in 10% neutral-buffered formalin and specimens for transmission electron microscopy were processed separately. For biochemical assays, renal tissue was homogenized in 50 mmol/L Tris–HCl buffer (pH 7.4). Homogenates were clarified by centrifugation at 10,000× *g* for 15 min at 4 °C, and the recovered supernatants were stored at −80 °C until assay.

### 2.6. Evaluation of Kidney Function

Serum creatinine, urea, and uric acid were measured using commercial colorimetric kits (Bio Diagnostics Co., Giza, Egypt). Creatinine was determined by the kinetic Jaffé method at 495 nm, urea by the urease-Berthelot method at 530–560 nm, and uric acid by a uricase-peroxidase assay at 510 nm [36]. Kidney injury molecule-1 (Kim-1) in kidney homogenates was quantified by ELISA (MyBioSource, San Diego, CA, USA; Cat. No. MBS762913) with 450 nm absorbance read.

### 2.7. Assessment of Renal Redox Status and Oxidative Injury

Renal homogenates were analyzed for reduced glutathione (GSH) content and the activities of superoxide dismutase (SOD), catalase (CAT), and glutathione peroxidase (GPx) using colorimetric assay kits from BioDiagnostic (Giza, Egypt). The respective catalog numbers were 2511 for GSH, 2521 for SOD, 2517 for CAT, and 2524 for GPx. Measurements were performed in accordance with the supplier protocols. Absorbance readings were obtained at 412 nm for GSH, 560 nm for SOD, 510 nm for CAT, and 340 nm for GPx.

Lipid oxidative damage was assessed by measuring thiobarbituric acid-reactive substances (TBARS) as malondialdehyde (MDA) equivalents using a commercial kit (BioDiagnostic, Cat. No. MD 2529), with absorbance read at 532 nm. Protein oxidation was evaluated by measuring protein carbonyl content using a commercial ELISA kit (MyBioSource, San Diego, CA, USA; Cat. No. MBS760520) according to the manufacturer’s instructions.

Reactive oxygen species generation was assessed in fresh renal-cell suspensions with a DCFH-DA fluorescence assay (MyBioSource, San Diego, CA, USA; Cat. No. MBS2540517). After incubation with the probe and washing, fluorescence was measured at 488 nm excitation and 525 nm emission. Results were expressed as relative fluorescence units normalized to the control group.

Oxidative DNA damage was assessed by measuring 8-hydroxy-2′-deoxyguanosine (8-OHdG) in kidney homogenates with an ELISA kit (MyBioSource, San Diego, CA, USA; Cat. No. MBS732375). Absorbance was read at 450 nm, and concentrations were derived from the corresponding standard curve. Renal Nrf2 and HO-1 concentrations were measured by ELISA using MyBioSource kits (Cat. Nos. MBS3807961 and MBS2024438, respectively), following the manufacturers’ instructions [37,38].

### 2.8. Evaluation of Renal Inflammatory Biomarkers

Renal tumor necrosis factor-α (TNF-α), interleukin-1β (IL-1β), interleukin-6 (IL-6), NF-κB, cyclooxygenase-2 (COX-2), and TLR4 were quantified in tissue homogenates using rat ELISA kits (MyBioSource: TNF-α Cat. No. MBS282960; IL-1β MBS232385; IL-6 MBS2021530; NF-κB MBS287521; COX-2 MBS127898; TLR4 MBS161614). Absorbance was measured at 450 nm, and concentrations were determined from standard curves.

### 2.9. Assessment of Renal Apoptosis Biomarkers

Activities of caspase-3 and caspase-8 in renal homogenates were determined using fluorescence Apo-Alert kits (Clontech Laboratories, Inc., Mountain View, CA, USA; caspase-3 Cat. No. 630215; caspase-8 Cat. No. 630219) according to the manufacturer’s instructions. Fluorescence generated by the cleaved 7-amino-4-trifluoromethylcoumarin (AFC) substrate was measured at 400 nm excitation and 505 nm emission. Caspase activities were normalized to total protein concentration.

### 2.10. Quantitative Gene Expression Analysis

Renal total RNA was isolated, reverse-transcribed, and amplified as detailed previously [38]. Primer sequences are listed in Table S1. The amplification program comprised initial denaturation at 95 °C for 3 min, followed by 40 cycles of 95 °C for 15 s, 58–60 °C for 30 s, and 72 °C for 30 s. Melt-curve analysis from 65 to 95 °C was performed to assess amplicon specificity. Relative mRNA expression was calculated using the 2^−ΔΔCt^ method [39] with β-actin as the reference gene. MIQE 2 guidelines were followed, and each sample was analyzed in duplicate [40].

### 2.11. Immunohistochemical Detection of NRF2 and NF-κB

Renal paraffin sections were processed for Nrf2 and NF-κB p65 immunostaining. After xylene deparaffinization and graded-ethanol rehydration, heat-induced antigen retrieval was performed in 0.01 M citrate buffer (pH 6.0). Endogenous peroxidase activity was quenched with 3% hydrogen peroxide in methanol, followed by a 30 min blocking step with 5% bovine serum albumin.

Sections were exposed overnight at 4 °C to rabbit anti-Nrf2 (1:200; Abcam, Cambridge, UK; ab62352) or rabbit anti-NF-κB p65 (1:200; Abcam, Cambridge, UK; ab32536). After incubation with a biotinylated goat anti-rabbit secondary antibody (1:500; Abcam) for 30 min, the signal was developed with streptavidin–horseradish peroxidase and 3,3′-diaminobenzidine. Mayer’s hematoxylin was used as the counterstain before slide dehydration, clearing, and mounting.

An assessor blinded to treatment allocation acquired and analyzed the images. Five randomly selected renal cortical fields were examined per animal at 400× magnification. Nrf2 and NF-κB p65 staining were quantified as immunoreactive area (%) using ImageJ version 1.54 (National Institutes of Health, Bethesda, MD, USA). Field-level measurements were averaged to generate one value per animal for statistical analysis.

### 2.12. Histopathological Analysis

Kidney specimens designated for light microscopy were processed and stained with hematoxylin and eosin as previously described [41]. For each group, histological assessment included three rats, three sections per rat, and four randomly selected fields per section. The evaluator was unaware of treatment allocation. Renal lesions were graded on a four-level ordinal scale, from 0 (no detectable injury) to 3 (severe injury), according to the scoring criteria shown in Figure 1. Field and section scores were averaged within each animal, and the resulting animal-level score was used for statistical analysis.

### 2.13. Ultrastructural Evaluation of Kidney Tissues

Kidney samples were fixed in 2.5% glutaraldehyde in 0.1 M phosphate buffer at 4 °C for 24 h, washed, and post-fixed in 1% osmium tetroxide for 2 h. Tissues were dehydrated in graded ethanol, cleared in acetone, and embedded in epoxy resin. Ultrathin sections (60–70 nm) were cut with an ultramicrotome, stained with uranyl acetate and lead citrate, mounted on copper grids, and examined with a JEM-2100 transmission electron microscope (TEM; JEOL Ltd., Akishima, Tokyo, Japan) at 160 kV. Multiple fields per section were imaged for ultrastructural assessment.

### 2.14. Statistical Analysis

Data were analyzed using SAS software, version 9.3 (SAS Institute Inc., Cary, NC, USA; 2012) and are presented as mean ± SEM. The experimental unit was one individual rat. For biochemical and molecular analyses, seven rats per group were analyzed. For histopathological, ultrastructural, and immunohistochemical analyses, three rats per group were evaluated; where multiple sections or fields were assessed from an individual animal, values were averaged within that animal before group-level analysis. Normality and homogeneity of variance were assessed using the Shapiro–Wilk and Levene tests, respectively. When assumptions were satisfied, group differences were evaluated using one-way analysis of variance followed by Tukey’s honestly significant difference post hoc test. Differences were considered statistically significant at *p* < 0.05. Graphs were prepared using GraphPad Prism version 9.1.0, build 221 (San Diego, CA, USA).

## 3. Results

### 3.1. Physicochemical Characterization and In Vitro Release of PTS-PLGA

TEM showed well-defined, nearly spherical PTS-PLGA nanoparticles with intact morphology and no apparent aggregation (Figure 2A). The size distribution histogram showed particles predominantly within the 83–108 nm range (Figure 2B). Dynamic light scattering showed a mean hydrodynamic diameter of 153 nm and a PDI of 0.152, consistent with a relatively homogeneous particle-size distribution (Figure 2C). The zeta potential was −33.65 mV (Figure 2D). The formulation showed an entrapment efficiency of 81.73%.

FTIR spectra of the PTS-PLGA formulation displayed bands attributable to PTS and PLGA, including O–H stretching around 3400 cm^−1^, C–H stretching near 2950 cm^−1^, a strong ester C=O peak at approximately 1750 cm^−1^, aromatic C=C stretching near 1600 cm^−1^, and C–O/C–O–C vibrations between 1250–1100 cm^−1^ (Figure 2E). The spectral profile was consistent with incorporation of PTS into the PLGA formulation; no additional prominent bands were observed under the analytical conditions used.

In vitro release testing showed that free PTS reached approximately 75% release at 4 h and near-complete release by 12 h (Figure 2F). In contrast, PTS-PLGA showed an initial release phase followed by slower release, reaching approximately 78% at 12 h and approximately 99% at 48 h. Under the tested in vitro conditions, the PTS-PLGA formulation showed a more prolonged release profile than free PTS.

### 3.2. Effect of Cadmium Exposure and PTS-PLGA on Renal Function

Cd exposure was associated with significantly higher serum urea, creatinine, and uric acid concentrations than the control condition (Figure 3A–C). Both free PTS and PTS-PLGA significantly reduced these markers relative to the Cd group (adjusted *p* < 0.05). Direct comparison between the Cd+PTS and Cd+PTS-PLGA groups showed significantly lower serum urea (adjusted *p* = 0.0003) and creatinine (adjusted *p* = 0.0186) with PTS-PLGA, whereas the difference in uric acid was not significant (adjusted *p* = 0.5880). Serum urea, creatinine, and uric acid in the Cd+PTS-PLGA group were not significantly different from control values (adjusted *p* = 0.220, 0.967, and 0.677, respectively). Renal Kim-1 was significantly higher in Cd-exposed rats than in controls. Both free PTS and PTS-PLGA were associated with significantly lower Kim-1 values than Cd exposure alone, and direct comparison demonstrated significantly lower Kim-1 in the Cd+PTS-PLGA group than in the Cd+PTS group (adjusted *p* = 0.003), supporting a greater effect of PTS-PLGA for this endpoint (Figure 3D).

### 3.3. Effect of Cadmium Exposure and PTS-PLGA on Nrf2/HO-1 Pathway, Antioxidant Status, and Oxidative Stress Biomarkers

Cd exposure was associated with significant reductions in renal Nrf2 protein and mRNA levels compared with controls (Figure 4A,B). For Nrf2 protein, free PTS did not produce a significant increase relative to Cd (adjusted *p* = 0.087), whereas PTS-PLGA significantly increased Nrf2 protein levels (adjusted *p* = 0.0004). However, the direct difference between the Cd+PTS and Cd+PTS-PLGA groups did not reach statistical significance (adjusted *p* = 0.055). In contrast, Nrf2 mRNA was significantly increased by both free PTS (adjusted *p* = 0.003) and PTS-PLGA (adjusted *p* < 0.0001) relative to Cd, with PTS-PLGA producing a significantly greater increase than free PTS (adjusted *p* < 0.0001). Cd exposure was also associated with significantly lower HO-1 protein and mRNA levels than controls (Figure 4C,D). Free PTS did not significantly alter HO-1 protein or mRNA levels relative to Cd (adjusted *p* = 0.865 and 0.416, respectively), whereas PTS-PLGA significantly increased both HO-1 protein (adjusted *p* = 0.0046) and mRNA (adjusted *p* < 0.0001). Direct comparisons further showed significantly higher HO-1 protein (adjusted *p* = 0.029) and mRNA (adjusted *p* < 0.0001) levels in the Cd+PTS-PLGA group than in the Cd+PTS group.

Cd exposure significantly reduced renal GSH content and SOD, CAT, and GPx activities compared with controls (Figure 4E–H). Free PTS significantly increased GSH and SOD relative to Cd exposure (adjusted *p* = 0.007 and 0.013, respectively), but did not significantly alter CAT activity (adjusted *p* = 0.561); GPx activity was significantly increased (adjusted *p* = 0.0006). PTS-PLGA significantly increased all four antioxidant indices relative to Cd (adjusted *p* < 0.0001 for GSH, SOD, and GPx; *p* = 0.011 for CAT). Direct comparison between the Cd+PTS and Cd+PTS-PLGA groups showed significantly higher GSH and SOD with PTS-PLGA (adjusted *p* = 0.003 and 0.002, respectively), whereas the differences in CAT and GPx were not significant (adjusted *p* = 0.175 and 0.129, respectively).

Cd exposure was associated with significantly increased renal ROS, MDA, protein carbonyl, and 8-OHdG levels compared with controls (Figure 5A–D). Free PTS significantly reduced ROS, protein carbonyl, and 8-OHdG relative to Cd exposure (adjusted *p* < 0.0001 for each), whereas its effect on MDA did not reach statistical significance (adjusted *p* = 0.059). In contrast, PTS-PLGA significantly reduced all four oxidative-damage markers relative to Cd (adjusted *p* < 0.0001 for each). Direct comparison between the Cd+PTS and Cd+PTS-PLGA groups demonstrated significantly lower ROS (adjusted *p* < 0.0001), MDA (adjusted *p* < 0.0001), protein carbonyl (adjusted *p* = 0.0001), and 8-OHdG (adjusted *p* = 0.0004) levels with PTS-PLGA. Moreover, 8-OHdG in the Cd+PTS-PLGA group did not differ significantly from the control group (adjusted *p* = 0.955), whereas ROS, MDA, and protein carbonyl remained significantly different from control values (adjusted *p* = 0.018, 0.003, and 0.0001, respectively).

### 3.4. Effect of Cadmium Exposure and PTS-PLGA on TLR4/NF-Κb Pathway, Cox-2, and Pro-Inflammatory Cytokines

Free PTS significantly reduced TLR4 mRNA (adjusted *p* = 0.0001) but not TLR4 protein (adjusted *p* = 0.148) relative to Cd, whereas PTS-PLGA significantly reduced both TLR4 protein and mRNA (adjusted *p* < 0.0001 for both). Direct comparison between the Cd+PTS and Cd+PTS-PLGA groups showed significantly lower TLR4 protein (adjusted *p* = 0.0008) and mRNA (adjusted *p* < 0.0001) with PTS-PLGA. Both free PTS and PTS-PLGA significantly reduced NF-κB protein and mRNA relative to Cd exposure. However, the direct differences between Cd+PTS and Cd+PTS-PLGA were not significant for either NF-κB protein (adjusted *p* = 0.139) or mRNA (adjusted *p* = 0.3005). NF-κB protein and mRNA levels in the Cd+PTS-PLGA group did not differ significantly from controls (adjusted *p* = 0.996 and 0.819, respectively).

Cd exposure was also associated with increased COX-2 protein and mRNA levels (Figure 6E,F). Free PTS did not significantly reduce either COX-2 protein (adjusted *p* = 0.123) or mRNA (adjusted *p* = 0.626) relative to Cd, whereas PTS-PLGA significantly reduced both measures (adjusted *p* < 0.0001 for both). Direct comparisons showed significantly lower COX-2 protein (adjusted *p* < 0.0001) and mRNA (adjusted *p* = 0.0002) in the Cd+PTS-PLGA group than in the Cd+PTS group.

Cd exposure significantly increased renal TNF-α, IL-6, and IL-1β levels compared with controls (Figure 6G–I). Free PTS significantly reduced IL-6 and IL-1β (adjusted *p* = 0.008 and 0.001, respectively) but not TNF-α (adjusted *p* = 0.599) relative to Cd. In contrast, PTS-PLGA significantly reduced all three cytokines relative to Cd (adjusted *p* = 0.0001 for TNF-α and *p* < 0.0001 for IL-6 and IL-1β). Direct comparison with free PTS showed significantly lower TNF-α, IL-6, and IL-1β levels with PTS-PLGA (adjusted *p* = 0.001, 0.009, and 0.023, respectively). TNF-α and IL-1β levels in the Cd+PTS-PLGA group were not significantly different from controls (adjusted *p* = 0.511 and 0.965, respectively), while the difference for IL-6 narrowly failed to reach significance (adjusted *p* = 0.069).

### 3.5. Effect of Cadmium Exposure and PTS-PLGA on Apoptotic Markers

Cd exposure was associated with significantly increased renal caspase-3 and caspase-8 activities compared with controls (Figure 7A,B). Both free PTS and PTS-PLGA significantly reduced caspase-3 (adjusted *p* = 0.0030 and *p* < 0.0001, respectively) and caspase-8 (adjusted *p* = 0.029 and *p* < 0.0001, respectively) activities relative to Cd exposure. Direct comparison between the Cd+PTS and Cd+PTS-PLGA groups showed significantly lower caspase-3 (adjusted *p* = 0.035) and caspase-8 (adjusted *p* = 0.025) activities with PTS-PLGA. Caspase-3 activity in the Cd+PTS-PLGA group did not differ significantly from the control group (adjusted *p* = 0.936), whereas caspase-8 remained significantly different from control (adjusted *p* = 0.017).

At the mRNA level, Cd exposure was associated with a significantly increased Bax/Bcl-2 ratio and p53 expression compared with controls (Figure 7C,D). Both free PTS and PTS-PLGA significantly reduced the Bax/Bcl-2 ratio (adjusted *p* = 0.0005 and *p* < 0.0001, respectively) and p53 expression (adjusted *p* = 0.0120 and *p* < 0.0001, respectively) relative to Cd exposure. Direct comparisons demonstrated a significantly lower Bax/Bcl-2 ratio (adjusted *p* = 0.0004) and p53 expression (adjusted *p* = 0.0002) in the Cd+PTS-PLGA group than in the Cd+PTS group. Nevertheless, these protein/activity and transcript-level apoptosis-related markers support modulation of apoptosis-associated processes but do not independently establish selective activation or inhibition of a specific intrinsic or extrinsic apoptotic pathway.

### 3.6. Effect of Cadmium Exposure and PTS-PLGA on Kidney Histopathology

Control kidneys showed preserved glomerular and tubular morphology (Figure 8A). The PTS-alone and PTS-PLGA-alone groups showed morphology comparable to the control group (Figure 8B,C). Cd exposure was associated with histological alterations, including vascular congestion, periglomerular lymphocytic infiltration, and glomerular atrophy (Figure 8D). The Cd+PTS group showed less extensive histological alteration than the Cd group, whereas the Cd+PTS-PLGA group showed comparatively better preservation of glomerular and tubular morphology and less apparent vascular congestion (Figure 8E,F). Semi-quantitative damage scores were significantly higher in Cd-treated rats than in controls (adjusted *p* < 0.0001). Both free PTS and PTS-PLGA significantly reduced renal damage scores compared with Cd exposure alone (adjusted *p* < 0.0001 for both). However, direct comparison between the Cd+PTS and Cd+PTS-PLGA groups showed no significant difference in damage scores (adjusted *p* = 0.574), indicating that statistical superiority of PTS-PLGA over free PTS was not demonstrated for this histopathological endpoint (Figure 8G).

### 3.7. Effect of Cadmium Exposure and PTS-PLGA on Kidney Ultrastructure

In the control, PTS, and PTS-PLGA groups, proximal tubular epithelial cells showed preserved ultrastructural features, including elongated mitochondria, rounded euchromatic nuclei, dense apical microvilli, and intact basement membranes (Figure 9A–C). Cd exposure was associated with ultrastructural alterations, including basal cytoplasmic vacuolization, condensed heterochromatic nuclei, small or disrupted mitochondria, and shortened irregular microvilli (Figure 9D). The Cd+PTS and Cd+PTS-PLGA groups showed ultrastructural features that were less severely altered than those in the Cd group, including more preserved nuclear morphology, mitochondrial appearance, and apical microvilli. Minimal cytoplasmic vacuolization was observed in the Cd+PTS-PLGA group (Figure 9E,F).

### 3.8. Effect of Cadmium Exposure and PTS-PLGA on Nrf2 and NF-κB Immunoreactivity

Control, PTS-alone, and PTS-PLGA-alone kidneys showed comparable Nrf2 immunoreactivity in cortical tubular epithelial cells (Figure 10I-A–C). Cd exposure was associated with reduced Nrf2 immunoreactive area and weaker tubular staining (Figure 10I-D). Both free PTS and PTS-PLGA were associated with significantly higher Nrf2 immunoreactivity than Cd exposure alone (adjusted *p* = 0.0002 and *p* < 0.0001, respectively). Direct comparison between the treatment groups showed significantly higher Nrf2 immunoreactivity with PTS-PLGA than with free PTS (adjusted *p* = 0.002). Moreover, Nrf2 immunoreactivity in the Cd+PTS-PLGA group did not differ significantly from that in controls (adjusted *p* = 0.636). (Figure 10I-E–G). Because the analysis quantified immunoreactive area rather than nuclear and cytoplasmic staining separately, these observations describe overall Nrf2 immunoreactivity and do not establish Nrf2 nuclear translocation.

Control, PTS-alone, and PTS-PLGA-alone sections showed low NF-κB immunoreactivity (Figure 10II-A–C). Cd exposure was associated with increased NF-κB immunoreactive area in cortical tubules (Figure 10II-D). Both free PTS and PTS-PLGA were associated with significantly lower NF-κB immunoreactivity than Cd exposure alone (adjusted *p* < 0.0001 for both). However, direct comparison between the Cd+PTS and Cd+PTS-PLGA groups showed no significant difference in NF-κB immunoreactivity (adjusted *p* = 0.685), indicating that statistical superiority of PTS-PLGA over free PTS was not demonstrated for this endpoint (Figure 10II-E–G). These data describe altered NF-κB immunoreactivity; they do not directly establish NF-κB activation state or nuclear localization.

## 4. Discussion

Cadmium-induced nephrotoxicity involves oxidative injury, inflammation, and cell-death responses, with proximal tubular epithelial cells being particularly susceptible [2,42]. In the present rat model, Cd exposure was associated with renal functional impairment, increased renal injury and oxidative-stress markers, inflammatory and apoptosis-related changes, and histological and ultrastructural damage. Free PTS and, more consistently, the PTS-PLGA formulation, attenuated several of these Cd-associated changes. Because an empty-PLGA control was not included, the findings should be attributed to the tested PTS-PLGA formulation rather than exclusively to PTS or the PLGA carrier.

Elevated serum creatinine, urea, and uric acid in Cd-exposed rats are consistent with impaired renal excretory function [2]. Urea reflects protein catabolism and renal clearance [43], creatinine is commonly used as an index of glomerular filtration [44], and uric acid accumulation may accompany altered excretion and purine metabolism [42]. In this study, PTS-PLGA reduced these markers relative to Cd exposure, consistent with attenuation of Cd-associated renal dysfunction. However, changes in these serum markers should not be interpreted as direct measurements of glomerular filtration rate or tubular reabsorptive capacity.

Kim-1 is an established marker of proximal tubular injury [45]. The Cd-associated increase in Kim-1 and its reduction after treatment provide additional evidence consistent with tubular protection. PTS-PLGA showed a larger improvement than free PTS for Kim-1; however, claims of associated greater improvement under the present dosing conditions should be limited to endpoints for which a direct PTS-PLGA versus free-PTS comparison was statistically significant. Previous work has reported renoprotective effects of PTS in other experimental settings, including renal ischemia–reperfusion injury and hyperuricemic nephropathy [23,24]. To our knowledge, this study provides evidence in a Cd-induced rat nephrotoxicity model, although this claim should be rechecked through a final targeted literature search immediately before submission.

The greater effects observed with PTS-PLGA than with free PTS for selected endpoints may reflect differences in formulation and release behavior. The present in vitro release data support sustained PTS release from the PTS-PLGA formulation; however, pharmacokinetics, systemic bioavailability, cellular uptake, and renal biodistribution were not measured. Therefore, these potential explanations should be considered hypotheses rather than established mechanisms [27,28,29].

Cd toxicity is closely linked to oxidative stress. Increased ROS can damage lipids, proteins, and nucleic acids, contributing to renal cell injury and dysfunction [4,14]. In the present study, Cd increased renal ROS, MDA, protein carbonyls, and 8-OHdG, supporting concurrent lipid peroxidation, protein oxidation, and oxidative DNA damage [15,46,47,48]. Cd also reduced GSH content and SOD, CAT, and GPx activities, indicating compromised antioxidant defenses. GSH contributes to intracellular redox homeostasis [49], CAT detoxifies hydrogen peroxide [50], SOD catalyzes superoxide dismutation [51], and GPx reduces hydrogen peroxide and lipid hydroperoxides using GSH [52]. PTS and PTS-PLGA improved several antioxidant and oxidative-damage indices, with the nanoparticle formulation generally producing greater changes. These findings are consistent with an antioxidant-associated effect of the formulation, but they do not identify the direct molecular source of reduced ROS or establish a single antioxidant mechanism. Prior work supports antioxidant properties of PTS [53,54].

Nrf2 is a redox-sensitive transcription factor that can regulate antioxidant-response genes, including HO-1. Under oxidative conditions, Nrf2 dissociates from Keap1 and translocates to the nucleus, where it interacts with antioxidant-response elements [18,55]. HO-1 is an inducible cytoprotective enzyme that degrades heme into biliverdin, carbon monoxide, and free iron [56]. In the present study, Cd was associated with downregulated renal Nrf2 and HO-1 mRNA and ELISA-detected protein levels, whereas PTS-PLGA was associated with increments in Nrf2/HO-1-related markers. These results are consistent with modulation of an antioxidant-response pathway in Cd-exposed kidneys. However, total tissue mRNA/protein assays and immunohistochemical reactive-area analysis do not directly demonstrate Nrf2 nuclear translocation, ARE binding, or Nrf2-dependent transcriptional activity. Accordingly, the results should not be interpreted as definitive evidence that Nrf2 activation mediates the protective effects of PTS-PLGA. The broader literature indicates that toxic-metal exposure can disrupt Nrf2 signaling, although the direction and magnitude of the response may depend on the dose, tissue, timing, and assay used [57]. PTS has been reported to influence Nrf2-related responses in other experimental systems [58].

The available data support a coordinated oxidative stress-defense-injury framework but do not resolve the temporal sequence of these events. Cd-associated ROS generation and depletion of GSH and antioxidant-enzyme activities may increase lipid, protein, and DNA oxidation, promote mitochondrial dysfunction, and facilitate inflammatory and apoptosis-related signaling [59]. In parallel, Nrf2/HO-1 functions as a redox-responsive cytoprotective system that can be induced in response to oxidative or electrophilic stress and can limit subsequent cellular injury [19]. Thus, the lower oxidative-damage markers and apoptosis-related indices observed after PTS or PTS-PLGA treatment may result from reduced oxidant burden, enhanced endogenous antioxidant defense, or a combination of both processes. Although the changes in Nrf2/HO-1-related markers are consistent with support of an antioxidant-response pathway, the present endpoint measurements do not establish whether PTS-PLGA initially suppressed ROS generation, primarily activated Nrf2/HO-1 signaling, or acted through both mechanisms. Time-resolved studies, direct evaluation of Nrf2 nuclear translocation and transcriptional activity, and pharmacological or genetic disruption of Nrf2/HO-1 signaling will be necessary to define the causal sequence and determine whether this pathway mediates the anti-apoptotic effects of the formulation.

Inflammation is another important component of Cd nephrotoxicity. The increased renal TNF-α, IL-1β, IL-6, TLR4, NF-κB, and COX-2-related markers observed after Cd exposure are consistent with an inflammatory response [16,60]. TLR4-related signaling can contribute to inflammatory responses through downstream NF-κB-regulated genes, including cytokines and COX-2 [61,62,63]. Both PTS and PTS-PLGA reduced selected inflammatory markers, with more consistent changes in the PTS-PLGA group. These results are consistent with reduced inflammatory signaling, but they do not prove direct TLR4 inhibition, NF-κB p65 phosphorylation changes, or inhibition of NF-κB nuclear translocation. Such claims would require activation-state assays, subcellular fractionation, or appropriately quantified nuclear-localization analysis. Therefore, the observed changes are best described as reduced TLR4/NF-κB-related inflammatory markers.

Oxidative injury and inflammatory responses can be accompanied by apoptosis. Increased caspase-3 activity is consistent with activation of executioner caspase activity, while increased caspase-8 activity is compatible with death-receptor-related apoptotic signaling [64,65]. Cd-associated apoptosis has been reported in renal injury models [14,66]. p53 may promote apoptosis in response to DNA damage, in part through regulation of pro-apoptotic proteins such as Bax [67,68], whereas Bcl-2 contributes to mitochondrial integrity and anti-apoptotic signaling [69,70]. The Cd-associated increases in caspase-3/caspase-8 activities, p53 expression, and the Bax/Bcl-2 mRNA ratio, together with their improvement after PTS or PTS-PLGA treatment, are consistent with attenuation of apoptosis-related changes that may occur downstream of reduced oxidative injury and/or enhanced cellular stress-defense responses [71,72]. Nevertheless, the available data do not distinguish intrinsic from extrinsic apoptosis definitively and do not directly measure mitochondrial membrane potential, cytochrome c release, or protein-level Bax and Bcl-2 expression. Thus, PTS-PLGA should be described as being associated with attenuation of apoptosis-related markers, rather than as proven to inhibit intrinsic and extrinsic apoptotic pathways.

Histopathological and ultrastructural findings support the biochemical and molecular observations. Cd exposure produced renal structural injury, including tubular, glomerular, and ultrastructural changes documented in this study, while free PTS and PTS-PLGA were associated with improved tissue architecture. The concordance between biochemical injury markers, blinded histological/IHC assessment, and TEM observations strengthens the evidence for a formulation-associated renoprotective effect. In line with the molecular data, the Nrf2 immunoreactive area was lower, and the NF-κB immunoreactive area was higher in Cd-exposed kidneys, whereas PTS-PLGA partially or substantially reversed these changes. Because IHC quantification assessed reactive area rather than separate nuclear and cytoplasmic localization, these findings demonstrate altered immunoreactivity but not nuclear transcription-factor activation or translocation.

The relevance of the above findings may extend beyond Cd exposure, but such extrapolation should remain mechanistically rather than therapeutically framed. Oxidative stress, mitochondrial dysfunction, inflammatory signaling, and apoptosis are shared features of several toxic nephropathies, including those associated with lead, mercury, arsenic, chromium, and certain nephrotoxic drugs [73,74]. Toxic metals can disrupt mitochondrial membrane potential and electron transport, promote reactive oxygen species generation, impair antioxidant defenses, and trigger inflammatory and apoptotic injury in tubular cells [74,75,76]. Thus, the concurrent modulation of Nrf2/HO-1, TLR4/NF-κB, and apoptosis-related markers by PTS-PLGA may be relevant to a broader set of kidney-injury mechanisms. However, the present findings demonstrate protection only in the Cd-exposed rat model and do not establish efficacy against other metals, chemical toxicants, or drug-induced nephropathies. These possibilities require direct evaluation in appropriate exposure-specific models.

The timing of administration should also be interpreted in the context of the experimental model. PTS and PTS-PLGA were administered 1 h before each CdCl_2_ injection to standardize dosing and evaluate whether repeated administration could attenuate renal injury during ongoing toxicant exposure. This design provides proof of concept for an adjunctive protective strategy during predictable or repeated exposure, rather than evidence for a broadly applicable preventive intervention. Such circumstances may include occupational settings with potential Cd exposure, including battery manufacture or recycling, metal smelting, welding or soldering, electroplating, pigment production, and mining-related activities. Occupational Cd exposure can occur through inhalation of contaminated dusts, fumes, or mists, whereas environmental exposure may occur through contaminated food, water, dust, and tobacco smoke. However, these situations should not be interpreted as an indication for routine prophylactic use of PTS-PLGA in exposed workers. The primary prevention of Cd-related disease remains reduction or elimination of exposure through substitution where feasible, engineering and work-practice controls, exposure monitoring, and appropriate personal protective equipment [77,78]. Whether PTS-PLGA is beneficial when initiated after exposure, administered intermittently, or used under clinically practical dosing schedules remains unknown and should be investigated in future studies.

It is worth noting that the more favorable outcomes observed in the current PTS-PLGA group should be interpreted cautiously. Free PTS and PTS-PLGA were administered at a nominal PTS-equivalent dose calculated using the formulation’s encapsulation efficiency, but circulating PTS concentrations, renal drug exposure, and biodistribution were not determined. Consequently, the present design does not establish pharmacokinetic equivalence between formulations or demonstrate kidney-specific delivery. The observed differences may reflect altered release, stability, absorption, systemic exposure, tissue distribution, or other formulation-dependent factors. Dedicated pharmacokinetic and biodistribution studies, together with an empty-PLGA nanoparticle control, are required to define the mechanism underlying the apparent advantage of PTS-PLGA.

Several formulation-related mechanisms may plausibly contribute to the more consistent effects observed with PTS-PLGA. First, the sustained in vitro release profile indicates that PLGA encapsulation may prolong PTS availability relative to the rapidly released free compound. Second, encapsulation may protect PTS from degradation and improve its apparent dispersion in the gastrointestinal environment, potentially modifying absorption after oral administration. Third, nanoparticle formulation may alter the temporal pattern of systemic and tissue exposure, which could influence the extent and duration of antioxidant, anti-inflammatory, and apoptosis-related responses during repeated Cd challenge. However, these mechanisms were not directly evaluated in the present study. The results therefore do not demonstrate enhanced oral bioavailability, increased renal PTS exposure, renal nanoparticle accumulation, or kidney-targeted delivery. These possibilities require direct comparison of free PTS and PTS-PLGA through plasma pharmacokinetic analyses, tissue PTS quantification, and carrier biodistribution studies.

The interpretation of formulation-related effects also requires consideration of the PLGA carrier itself. Although PLGA is widely used as a biodegradable polymer in drug-delivery systems, its general biocompatibility does not establish the in vivo disposition or safety of the specific PTS-PLGA formulation used here. Nanoparticle biodistribution, clearance, tissue retention, and potential toxicity may vary according to particle size, surface charge, composition, colloidal behavior, dose, and route of administration. These issues are particularly relevant in a renal injury model because nanoparticle handling may involve filtration, tubular uptake, secretion, or tissue retention. Accordingly, the present results do not establish whether PTS-PLGA, PLGA degradation products, or residual formulation components accumulated in the kidney or other organs, nor do they exclude a vehicle-related contribution to the observed responses.

This study has several limitations. First, empty PLGA nanoparticles and Cd plus empty-PLGA controls were not included; therefore, the independent contribution of PLGA cannot be separated conclusively from that of PTS in the tested formulation. Accordingly, potential carrier-related renal or systemic effects, including effects associated with renal retention or extra-renal distribution, cannot be excluded. Second, only male rats were used, and sex-related differences in Cd toxicity and treatment responses were not evaluated. Third, a single Cd dose, exposure duration, and PTS regimen were assessed. The pre-exposure dosing schedule, in addition, was designed to test prophylactic activity during a controlled, repeated Cd challenge and does not establish efficacy when treatment is initiated after exposure or after renal injury has developed. Fourth, although PTS-PLGA displayed a sustained in vitro release profile, formulation-specific pharmacokinetics, renal biodistribution, tissue PTS concentrations, batch-to-batch reproducibility, and long-term systemic safety were not investigated. Renal and extra-renal Cd concentrations were also not quantified; therefore, the study cannot determine tissue Cd burden, confirm comparable tissue Cd exposure among groups, or establish whether free PTS or PTS-PLGA altered Cd absorption, distribution, retention, or elimination. Future studies should measure Cd concentrations in kidney, liver, blood, and urine using validated elemental-analysis methods. Future studies should therefore include empty-PLGA and Cd plus empty-PLGA control groups, as well as quantitative measurement of PTS and nanoparticle/carrier disposition in plasma, kidney, liver, spleen, and other relevant organs, coupled with repeated-dose systemic and renal safety assessment. Fifth, although renal mRNA expression, total protein measurements, and immunohistochemical reactive-area quantification indicated changes in Nrf2/HO-1-related and TLR4/NF-κB-related markers, these approaches do not establish functional pathway activation, inhibition, nuclear translocation, phosphorylation status, DNA-binding activity, or transcriptional activity. The absence of Nrf2 nuclear-localization or cytoplasmic/nuclear fractionation analyses, ARE-DNA-binding or reporter assays, NF-κB phosphorylation and nuclear-translocation assessment, and pharmacological or genetic pathway-intervention experiments precludes definitive mechanistic inference. Finally, this experimental rat model cannot by itself establish effectiveness in human Cd exposure scenarios. These limitations warrant cautious interpretation and support further carrier-control, pharmacokinetic, and mechanistic validation studies.

## 5. Conclusions

Cadmium exposure was associated with renal functional impairment, oxidative damage, inflammatory responses, apoptosis-related alterations, and histological and ultrastructural injury in rats. Both free PTS and PTS-PLGA attenuated Cd-associated changes. Under the present experimental conditions, PTS-PLGA produced significantly greater improvement than free PTS for selected renal functional, oxidative-stress, inflammatory, antioxidant, and apoptosis-related endpoints, whereas no statistically significant between-formulation difference was observed for other outcomes. Overall, PTS-PLGA was associated with improved renal function and injury markers, enhanced antioxidant defenses, reduced oxidative and inflammatory changes, lower apoptosis-related markers, and preservation of renal morphology. These findings support further preclinical evaluation of PLGA-encapsulated PTS as a candidate formulation for Cd-associated renal injury. However, the absence of empty-PLGA controls, formulation-specific pharmacokinetic and biodistribution data, and renal Cd-burden measurements precludes conclusions regarding carrier-independent effects, altered PTS exposure, kidney-targeted delivery, or changes in Cd disposition. In addition, the lack of Nrf2 nuclear-localization, NF-κB phosphorylation, DNA-binding, and pathway-intervention experiments precludes definitive conclusions regarding functional activation or inhibition of these signaling pathways.

## Figures and Tables

**Figure 1:**
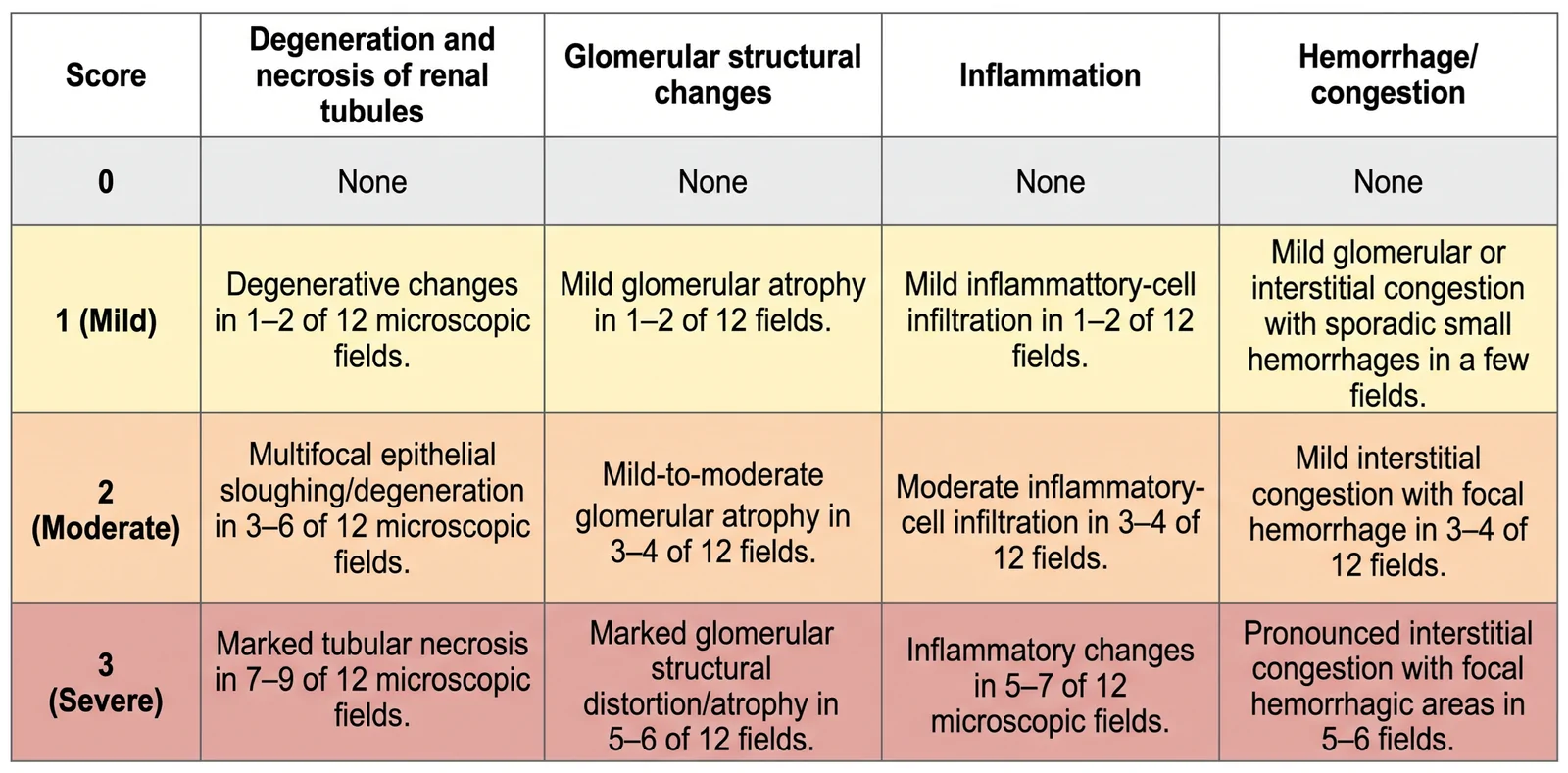
Semi-quantitative scoring system for renal histopathological injury. Renal tubular degeneration/necrosis, glomerular structural changes, inflammatory-cell infiltration, and hemorrhage/congestion were scored on an ordinal scale from 0 (none) to 3 (severe) across 12 prespecified microscopic fields per animal.

**Figure 2:**
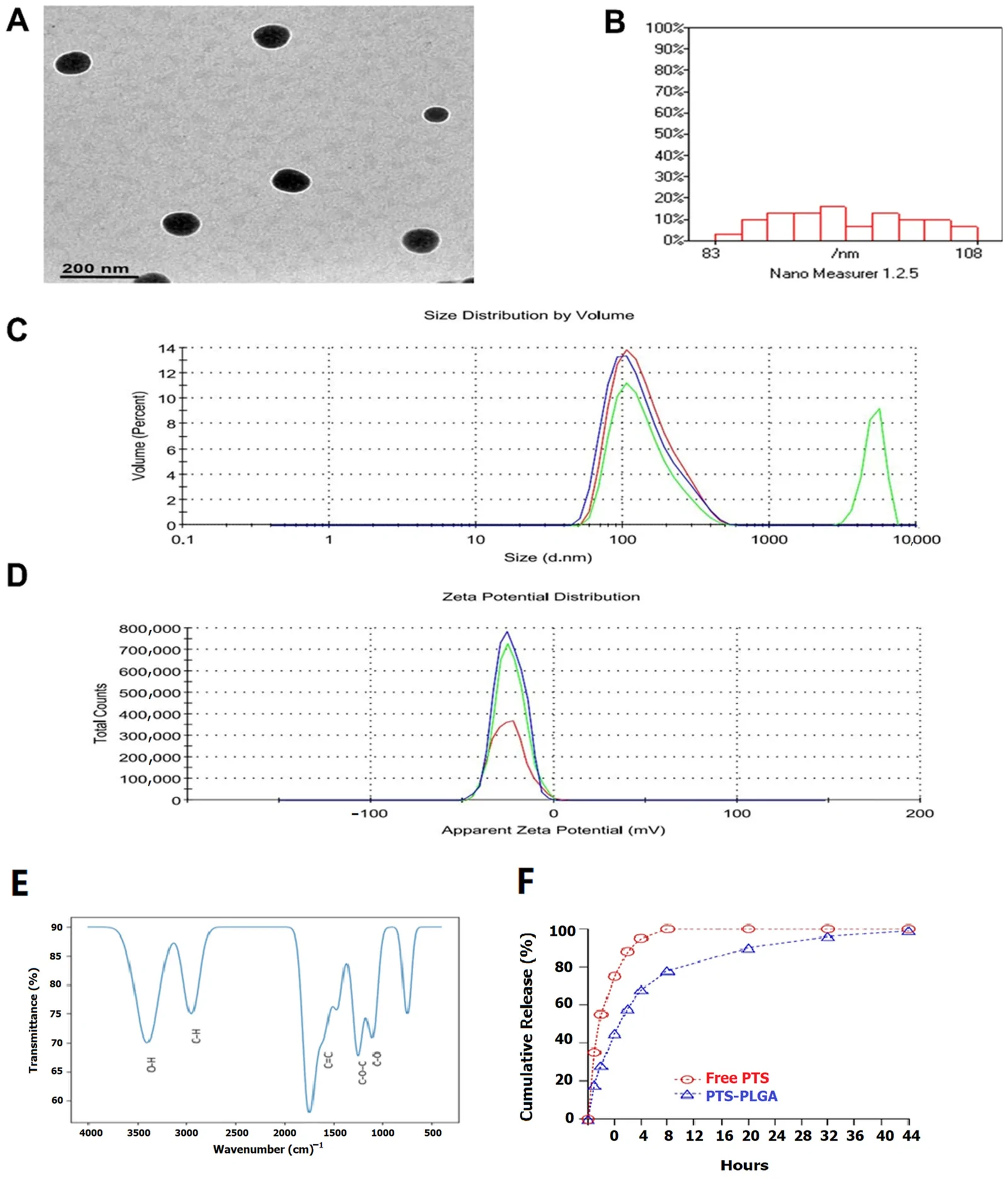
Physicochemical characterization and in vitro release profile of PTS-PLGA nanoparticles. (**A**) Representative TEM micrograph. (**B**) Particle-size distribution histogram. (**C**) Representative dynamic light-scattering (DLS) size distribution and mean hydrodynamic diameter (153 nm; PDI = 0.152) from replicate measurements (different colors). (**D**) Representative zeta-potential distributions (−33.65 mV) from replicate measurements. Colored traces represent individual measurement runs. (**E**) FTIR spectra of PTS, PLGA, and PTS-PLGA. (**F**) In vitro cumulative release profiles of free PTS and PTS-PLGA in PBS (pH 7.4) at 37 °C.

**Figure 3:**
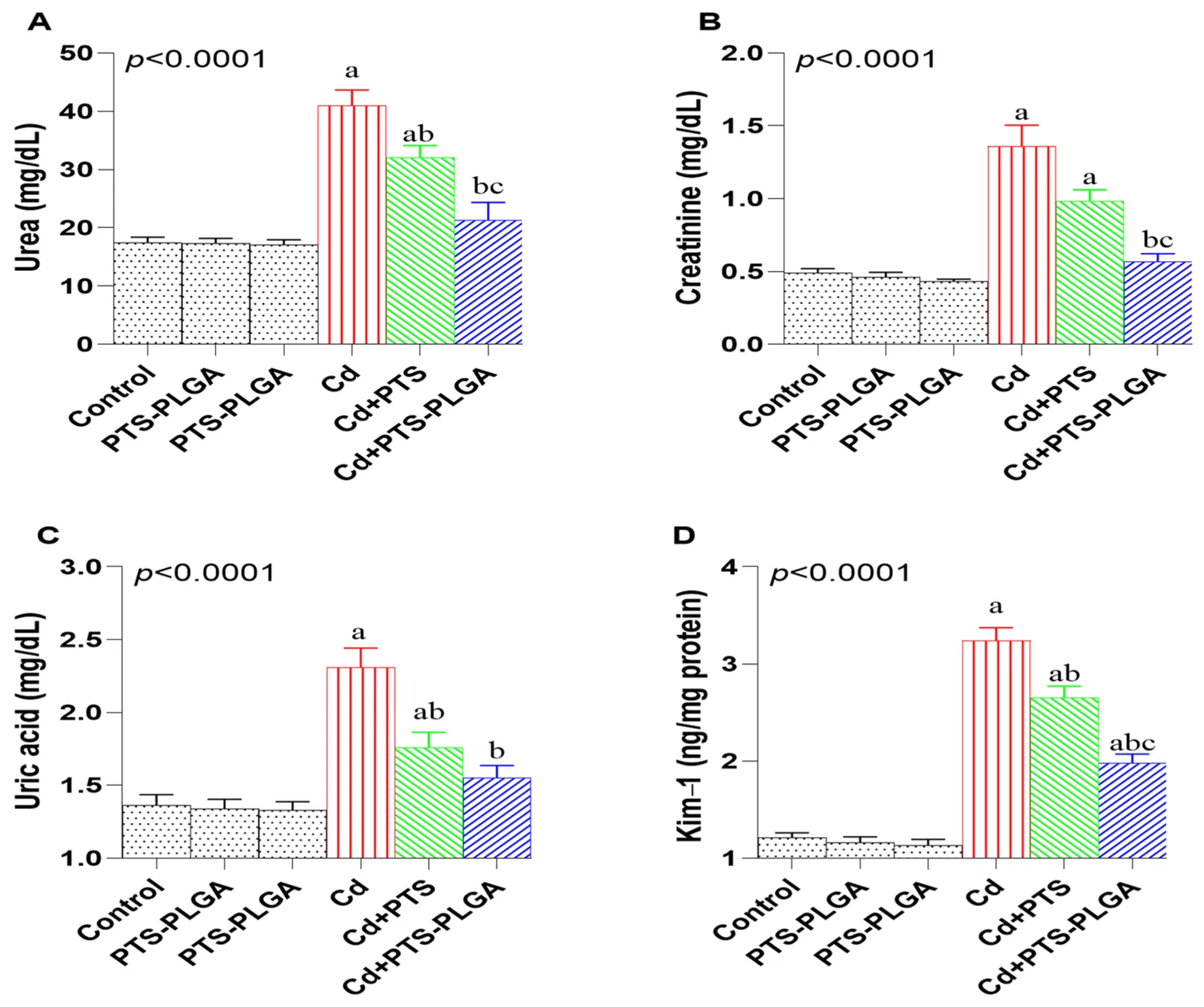
Effects of Cd exposure and treatment with free PTS or PTS-PLGA on renal function and injury markers. (**A**) Serum urea; (**B**) serum creatinine; (**C**) serum uric acid; and (**D**) renal Kim-1. Data are mean ± SEM (n = 7 biologically independent animals/group). One-way ANOVA followed by Tukey’s HSD test. a: *p* < 0.05 vs. control; b: *p* < 0.05 vs. Cd; c: *p* < 0.05 vs. Cd+PTS. Adjusted *p*-values from Tukey’s HSD post hoc comparisons for all assays presented in this figure are provided in Table S2.

**Figure 4:**
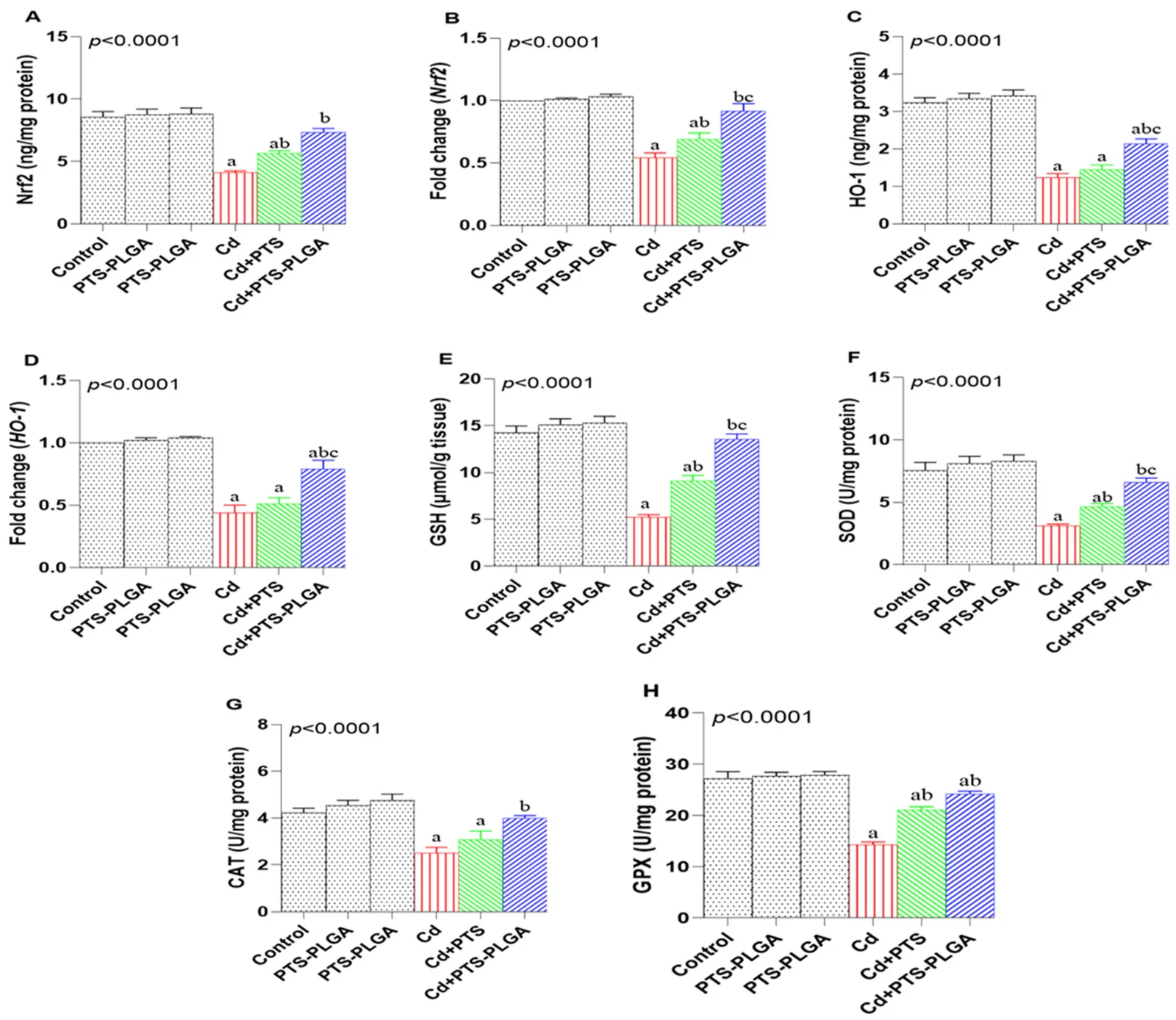
Effects of Cd exposure and treatment with free PTS or PTS-PLGA on Nrf2/HO-1-related markers and antioxidant indices. (**A**) Renal Nrf2 protein; (**B**) Nrf2 mRNA expression; (**C**) renal HO-1 protein; (**D**) HO-1 mRNA expression; (**E**) GSH; (**F**) SOD activity; (**G**) CAT activity; and (**H**) GPx activity. Data are the mean ± SEM (n = 7 biologically independent animals/group). One-way ANOVA followed by Tukey’s HSD test. a: *p* < 0.05 vs. control; b: *p* < 0.05 vs. Cd; c: *p* < 0.05 vs. Cd+PTS. This figure reports expression and activity measures; it does not directly assess Nrf2 nuclear localization or transcriptional activity. Adjusted *p*-values from Tukey’s HSD post hoc comparisons for all assays presented in this figure are provided in Table S2.

**Figure 5:**
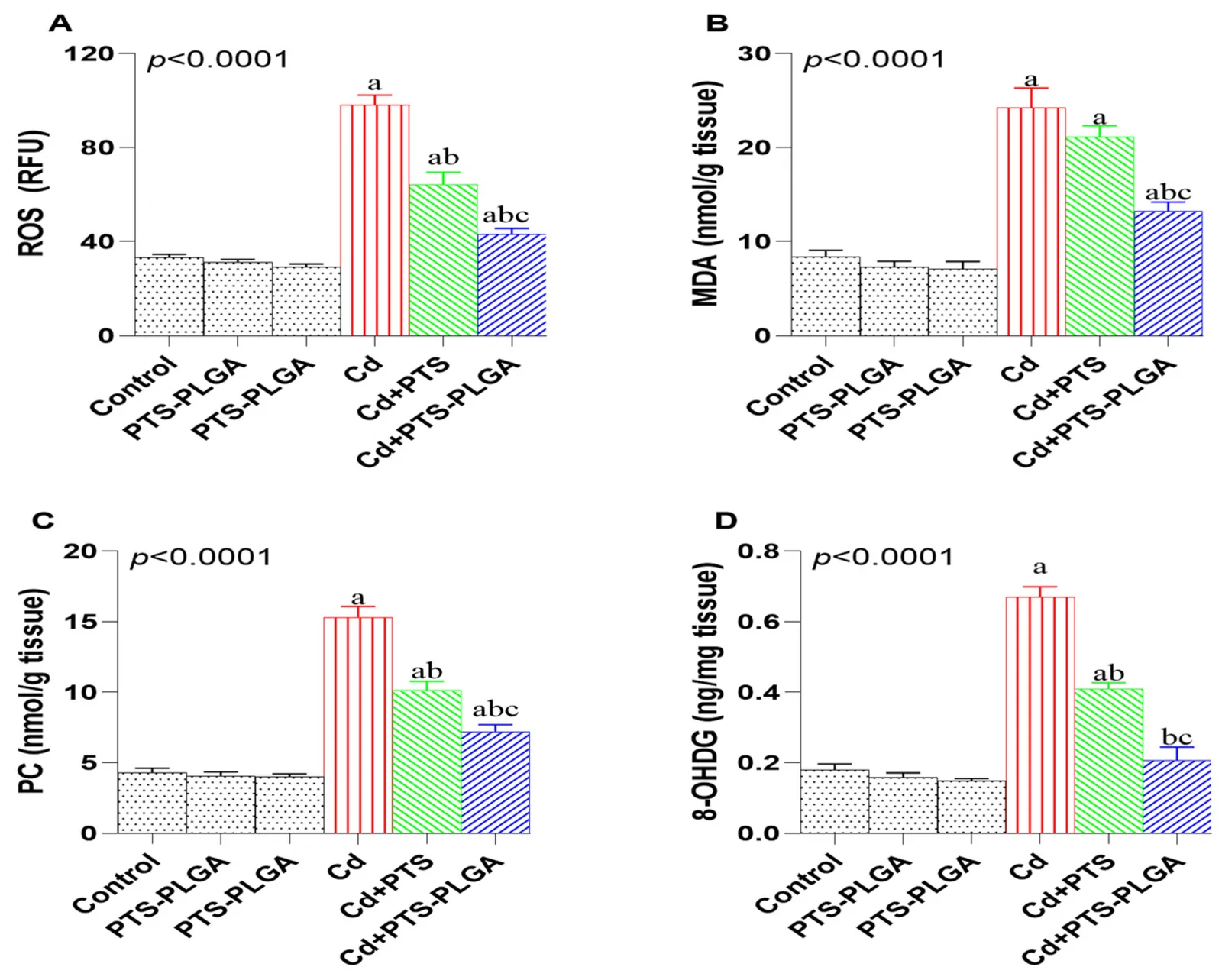
Effects of Cd exposure and treatment with free PTS or PTS-PLGA on renal oxidative-damage markers. (**A**) ROS; (**B**) MDA; (**C**) protein carbonyls; and (**D**) 8-OHdG. Data are mean ± SEM (n = 7 biologically independent animals/group). One-way ANOVA followed by Tukey’s HSD test. a: *p* < 0.05 vs. control; b: *p* < 0.05 vs. Cd; c: *p* < 0.05 vs. Cd+PTS. Adjusted *p*-values from Tukey’s HSD post hoc comparisons for all assays presented in this figure are provided in Table S3.

**Figure 6:**
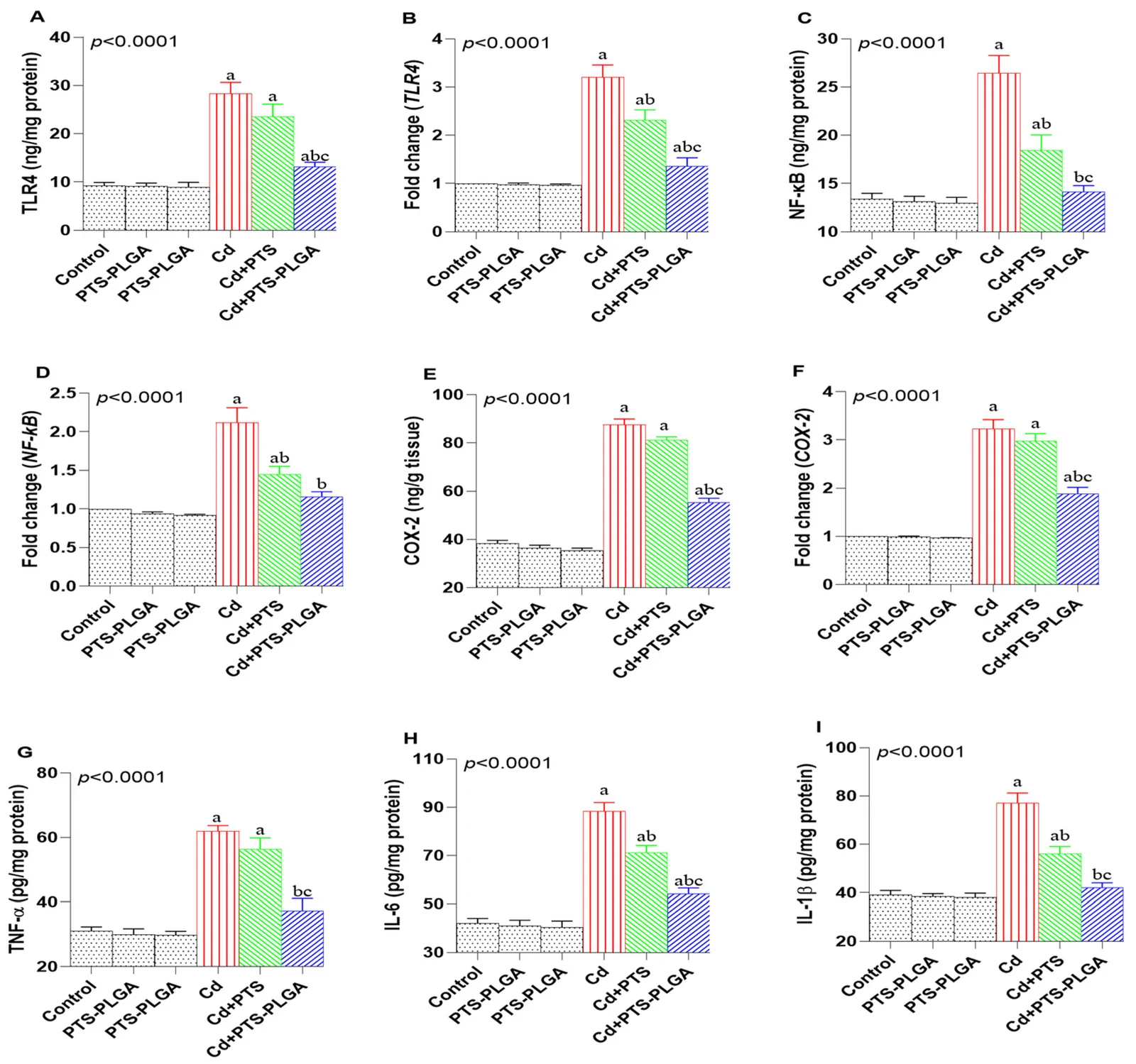
Effects of Cd exposure and treatment with free PTS or PTS-PLGA on renal TLR4/NF-κB-related, COX-2, and cytokine markers. (**A**) TLR4 protein; (**B**) TLR4 mRNA; (**C**) NF-κB protein; (**D**) NF-κB mRNA; (**E**) COX-2 protein; (**F**) COX-2 mRNA; (**G**) TNF-α; (**H**) IL-6; and (**I**) IL-1β. Data are mean ± SEM (n = 7 biologically independent animals/group). One-way ANOVA followed by Tukey’s HSD test. a: *p* < 0.05 vs. control; b: *p* < 0.05 vs. Cd; c: *p* < 0.05 vs. Cd+PTS. The measured markers provide evidence of altered inflammatory-marker expression; they do not directly measure TLR4 activity, NF-κB phosphorylation, DNA binding, or nuclear translocation. Adjusted *p*-values from Tukey’s HSD post hoc comparisons for all assays presented in this figure are provided in Table S3.

**Figure 7:**
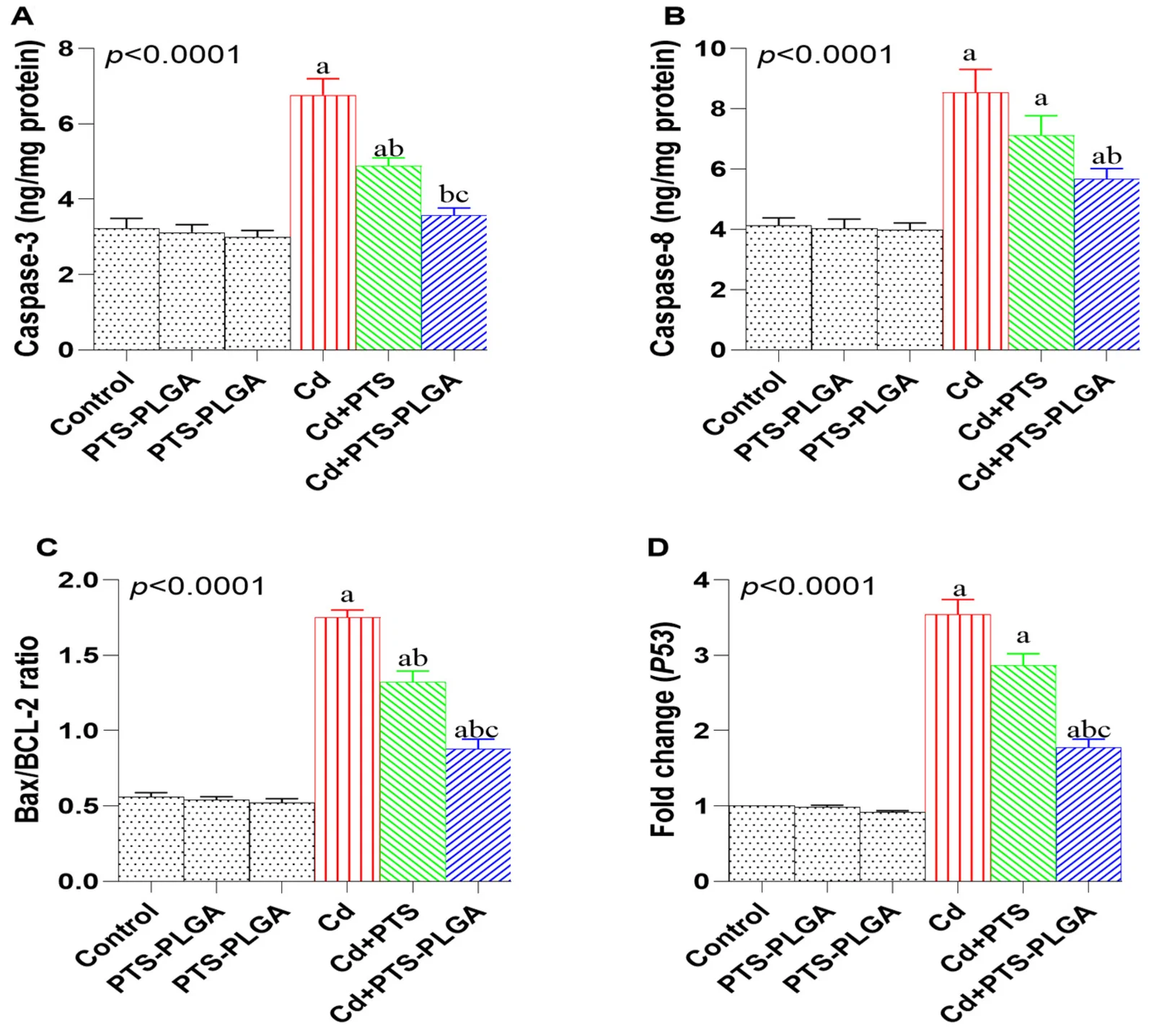
Effects of Cd exposure and treatment with free PTS or PTS-PLGA on renal apoptosis-related markers. (**A**) Caspase-3 activity; (**B**) caspase-8 activity; (**C**) Bax/Bcl-2 mRNA ratio; and (**D**) p53 mRNA expression. Data are mean ± SEM (n = 7 biologically independent animals/group). One-way ANOVA followed by Tukey’s HSD test. a: *p* < 0.05 vs. control; b: *p* < 0.05 vs. Cd; c: *p* < 0.05 vs. Cd+PTS. Adjusted *p*-values from Tukey’s HSD post hoc comparisons for all assays presented in this figure are provided in Table S4.

**Figure 8:**
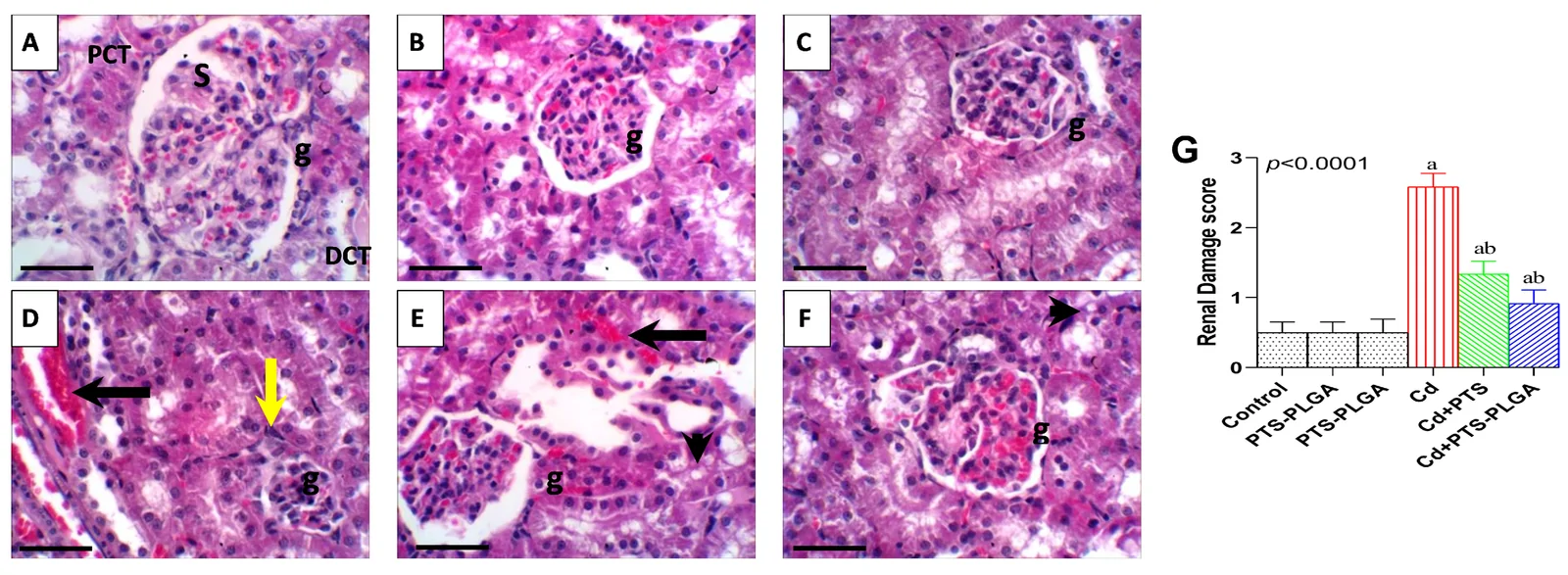
Renal histopathology in the experimental groups. Representative H&E-stained sections (×400) from (**A**) control, (**B**) PTS, (**C**) PTS-PLGA, (**D**) Cd, (**E**) Cd+PTS, and (**F**) Cd+PTS-PLGA groups. PCT, proximal convoluted tubules; DCT, distal convoluted tubules; g, glomerulus. Black arrow indicates a congested blood vessel; yellow arrow indicates inflammatory cells. Scale bar = 50 μm. (**G**) Semi-quantitative renal-injury scores. Data are mean ± SEM (n = 3 biologically independent animals/group). One-way ANOVA followed by Tukey’s HSD test. a: *p* < 0.05 vs. control; b: *p* < 0.05 vs. Cd. Adjusted *p*-values from Tukey’s HSD post hoc comparisons for renal damage severity scores are provided in Table S4.

**Figure 9:**
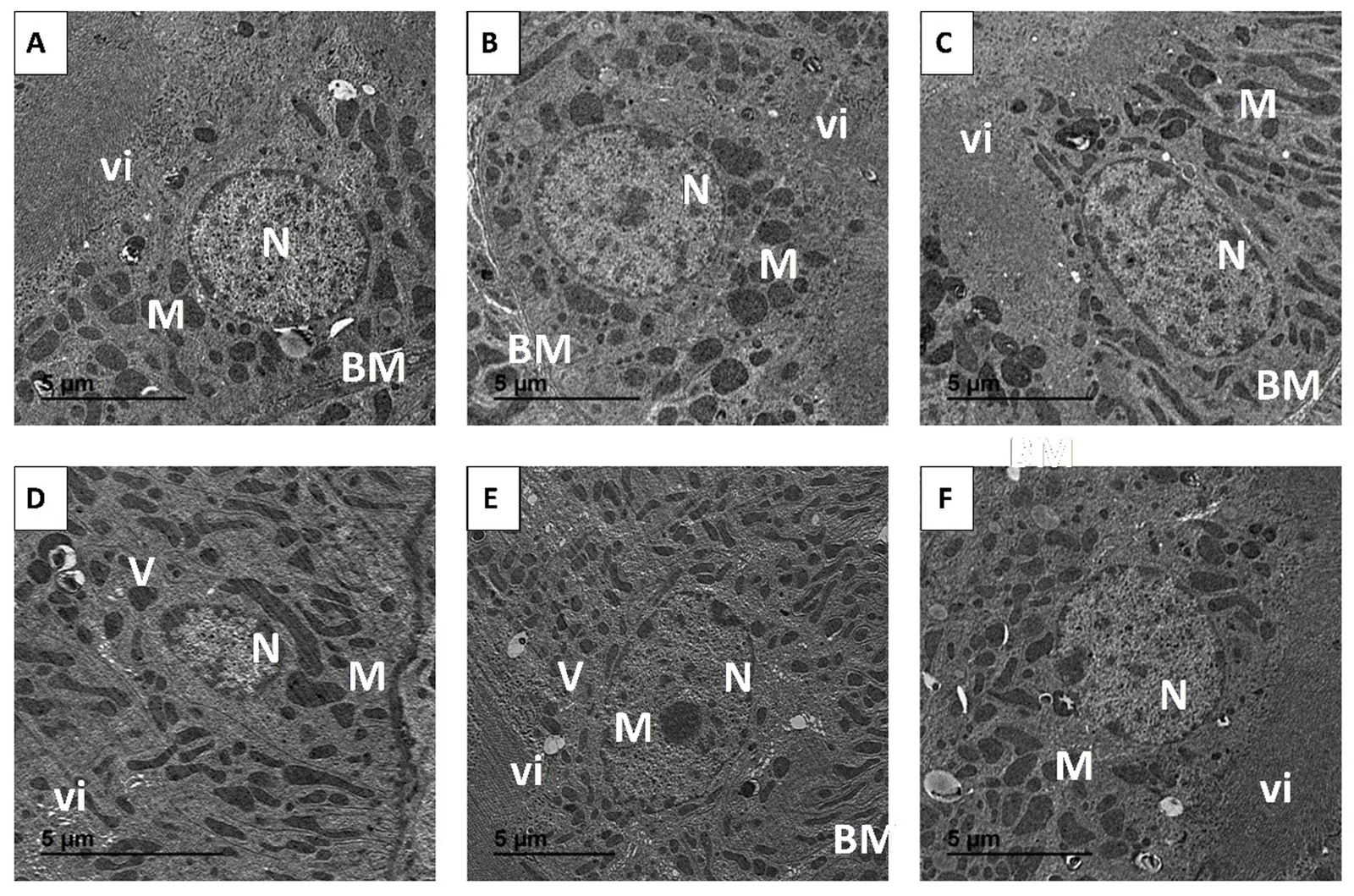
Representative TEM micrographs of renal cortex are shown from n = 3 biologically independent animals/group from (**A**) control, (**B**) PTS, (**C**) PTS-PLGA, (**D**) Cd, (**E**) Cd+PTS, and (**F**) Cd+PTS-PLGA groups. N, nucleus; M, mitochondria; BM, basement membrane; vi, microvilli.

**Figure 10:**
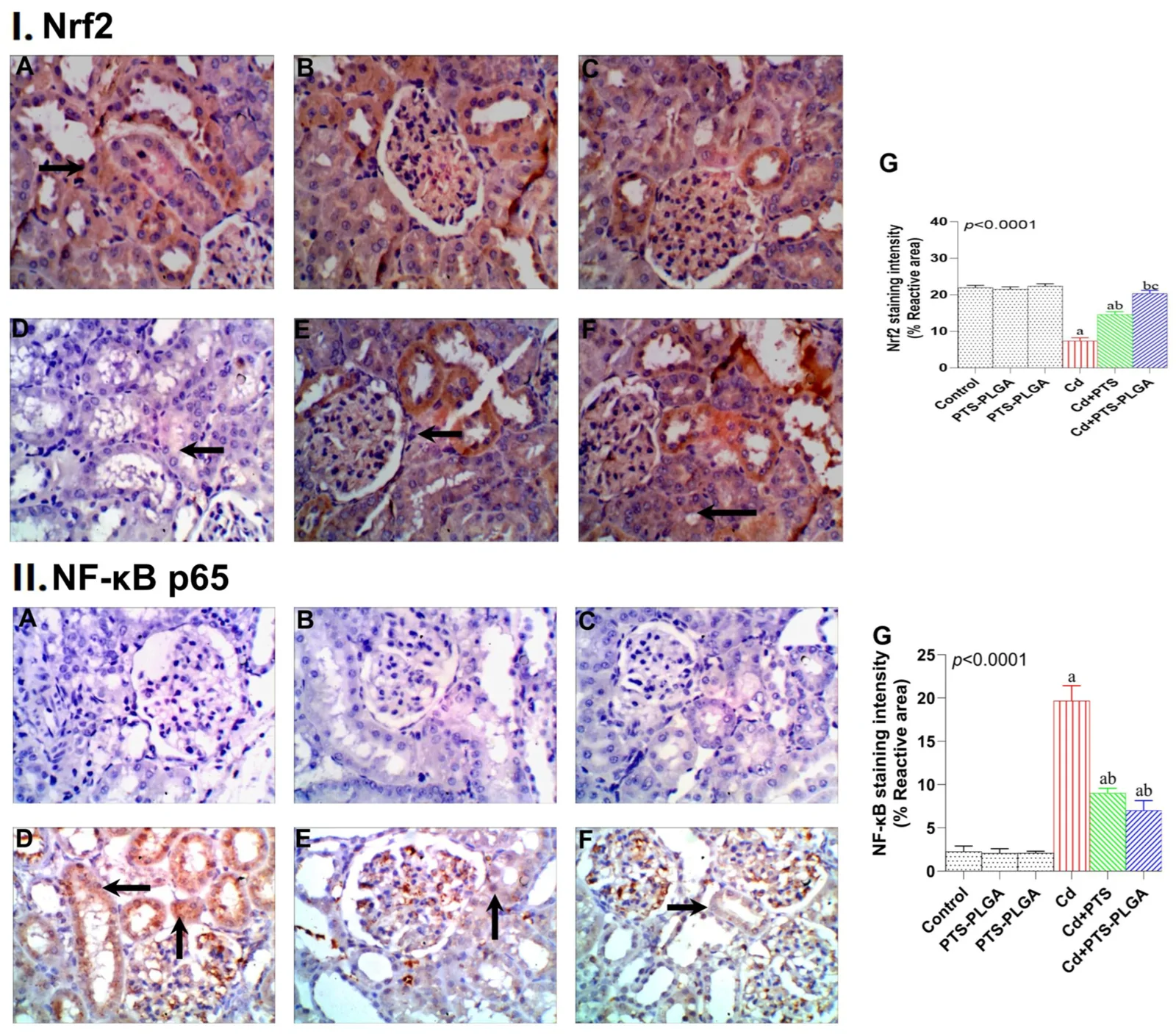
Immunohistochemical evaluation of Nrf2 and NF-κB p65 immunoreactivity in renal cortex. I, Nrf2: representative sections from (**A**) control, (**B**) PTS, (**C**) PTS-PLGA, (**D**) Cd, (**E**) Cd+PTS, and (**F**) Cd+PTS-PLGA groups; (**G**) quantitative Nrf2 immunoreactive area. II, NF-κB p65: representative sections from (**A**) control, (**B**) PTS, (**C**) PTS-PLGA, (**D**) Cd, (**E**) Cd+PTS, and (**F**) Cd+PTS-PLGA groups; (**G**) quantitative NF-κB immunoreactive area. Brown DAB staining indicates immunoreactivity. Black arrow indicates positive immunoexpression. Data are mean ± SEM (n = 3 biologically independent animals/group). One-way ANOVA followed by Tukey’s HSD test. a: *p* < 0.05 vs. control; b: *p* < 0.05 vs. Cd; c: *p* < 0.05 vs. Cd+PTS. Adjusted *p*-values from Tukey’s HSD post hoc comparisons for Nrf2 and NF-κB p65 immunoreactivity are provided in Table S4.

## Data Availability

The original contributions presented in this study are included in the article/Supplementary Material. Further inquiries can be directed to the corresponding author.

## Supplementary Materials

The following supporting information can be downloaded at https://www.mdpi.com/article/10.3390/toxics14100856/s1. Table S1: Sequences of primers utilized for gene expression analysis in kidney samples; Table S2: Adjusted *p*-values from Tukey’s HSD multiple-comparison test for renal function, injury, and antioxidant defense parameters following Cd exposure and treatment with free PTS or PTS-PLGA; Table S3: Adjusted *p*-values from Tukey’s HSD multiple-comparison test for renal oxidative damage and inflammatory parameters following Cd exposure and treatment with free PTS or PTS-PLGA; Table S4: Adjusted *p*-values from Tukey’s HSD multiple-comparison test for renal apoptosis-related parameters, histopathological damage scores, and Nrf2 and NF-κB immunoreactivity following Cd exposure and treatment with free PTS or PTS-PLGA.

## Abbreviations

The following abbreviations are used in this manuscript:

8-OHdG	8-Hydroxy-2′-deoxyguanosine
AFC	7-Amino-4-trifluoromethylcoumarin
ANOVA	Analysis of variance
ARE	Antioxidant response element
Bax	Bcl-2-associated X protein
Bcl-2	B-cell lymphoma 2
BSA	Bovine serum albumin
CAT	Catalase
Cd	Cadmium
CdCl_2_	Cadmium chloride
COX-2	Cyclooxygenase-2
DAB	3,3′-Diaminobenzidine
DCFH-DA	2′,7′-Dichlorodihydrofluorescein diacetate
DCT	Distal convoluted tubule
DLS	Dynamic light scattering
DNA	Deoxyribonucleic acid
EE	Entrapment efficiency
ELISA	Enzyme-linked immunosorbent assay
FTIR	Fourier-transform infrared spectroscopy
GSH	Reduced glutathione
GPx	Glutathione peroxidase
H&E	Hematoxylin and eosin
HO-1	Heme oxygenase-1
HRP	Horseradish peroxidase
IHC	Immunohistochemistry
IL-1β	Interleukin-1 beta
IL-6	Interleukin-6
Kim-1	Kidney injury molecule-1
Keap1	Kelch-like ECH-associated protein 1
MDA	Malondialdehyde
NF-κB	Nuclear factor kappa B
Nrf2	Nuclear factor erythroid 2-related factor 2
PCT	Proximal convoluted tubule
PC	Protein carbonyls
PDI	Polydispersity index
PLGA	Poly(lactic-co-glycolic acid)
PTS	Pterostilbene
PTS-PLGA	Pterostilbene-loaded poly(lactic-co-glycolic acid) nanoparticles
PBS	Phosphate-buffered saline
qRT-PCR	Quantitative reverse-transcription polymerase chain reaction
RFUs	Relative fluorescence units
RNA	Ribonucleic acid
ROS	Reactive oxygen species
SEM	Standard error of the mean
SOD	Superoxide dismutase
TEM	Transmission electron microscopy
TLR4	Toll-like receptor 4
TNF-α	Tumor necrosis factor alpha
