# Supplementary material for: PLGA-Encapsulated Pterostilbene Mitigates Cadmium-Induced Nephrotoxicity in Rats by Modulating Oxidative Stress, Inflammation, and Apoptosis
Source: Toxics. 2026 Sep 26;14(10):856. doi: 10.3390/toxics14100856 (PMC13709532; doi:10.3390/toxics14100856)
Supplement: Supplementary file 1 [file toxics-14-00856-s001.zip › toxics-4541719-supplementary.pdf]

PLGA-Encapsulated Pterostilbene Mitigates Cadmium-Induced Nephrotoxicity in Rats by Modulating Oxidative Stress, Inflammation, and Apoptosis

Table S1. Sequences of primers utilized for gene expression analysis in kidney samples.

Gene	Sense (5′-3′)	Antisense (5′-3′)
Nrf2	F: TTTGTAGATGACCATGAGTCG	R: TCCTGCCAAACTTGCTCCAT
HO-1	F: ATGTCCCAGGATTTGTCCGA	R: ATGGTACAAGGAGGCCATCA
NFκB	F: AGTCCCGCCCCTTCTAAAAC	R: CAATGGCCTCTGTGTAGCCC
TLR4	F: ATCATCCAGGAAGGCTTCCA	R: GCTGCCTCAGCAAGGACTTC
Cox-2	F: TGATGCCTTTGG AGT ATG GT	R: CAG GATGGGTTTGAG TCA
Bax	F: TTTCATCCAGGATCGAGCAG	R: AATCATCCTCTGCAGCTCCA
Bcl2	F: GACTTTGCAGAGATGTCCAG	R: TCAGGTACTCAGTCATCCAC
p53	F: CCTCAGCATCTTATCCGAGTGG	R: TGGATGGTGGTACAGTCAGAGC
β-Actin	F: CAGCCTTCCTTCTTGGGTATG	R: AGCTCAGTAACAGTCCGCCT

Nrf2 (nuclear factor erythroid 2–related factor 2), *HO-1* (heme oxygenase-1), *NFκB* (nuclear factor kappa B), TLR4 (toll-like receptor 4), *Cox-2* (cyclooxygenase-2), *Bax* (Bcl-2–associated X protein), *Bcl2* (B-cell lymphoma 2), *p53* (tumor protein p53), and *β-Actin* (beta-actin, used as a housekeeping gene for normalization).

Table S2. Adjusted *p*-values from Tukey’s HSD multiple-comparison test for renal function, injury, and antioxidant defense parameters following Cd exposure and treatment with free PTS or PTS-PLGA.

Groups / Assay	Urea	Creatinine	Uric acid	Kim-1	Nrf2 ElISA	Nrf2 mRNA	HO-1 ElISA	HO-1 mRNA	GSH	SOD	CAT	GPX
Tukey's multiple comparisons test	APV	APV	APV	APV	APV	APV	APV	APV	APV	APV	APV	APV
Control vs. PTS-PLGA	>0.9999	0.9997	>0.9999	0.9986	0.9984	0.9993	0.9902	0.9920	0.9027	0.6554	0.9301	0.9986
Control vs. PTS-PLGA	>0.9999	0.9908	0.9999	0.9878	0.9946	0.9079	0.9056	0.8645	0.8045	0.3610	0.6448	0.9904
Control vs. Cd	<0.000	<0.000	<0.000	<0.000	<0.000	<0.000	<0.000	<0.000	<0.000	<0.000	0.0032	<0.000
Control vs. Cd+PTS	<0.000	0.0051	0.0729	<0.000	0.0010	<0.000	<0.000	<0.000	0.0008	<0.000	0.0539	0.0015
Control vs. Cd+PTS-PLGA	0.2207	0.9677	0.6777	0.0008	0.2203	0.1271	0.0009	0.0008	0.9645	0.1661	0.9743	0.1492
PTS-PLGA vs. PTS-PLGA	>0.9999	0.9997	>0.9999	0.9999	>0.9999	0.9822	0.9977	0.9920	0.9999	0.9929	0.9880	>0.9999
PTS-PLGA vs. Cd	<0.000	<0.000	<0.000	<0.000	<0.000	<0.000	<0.000	<0.000	<0.000	<0.000	0.0007	<0.000
PTS-PLGA vs. Cd+PTS	<0.000	0.0032	0.0562	<0.000	0.0006	<0.000	<0.000	<0.000	0.0002	<0.000	0.0112	0.0009
PTS-PLGA vs. Cd+PTS-PLGA	0.2013	0.8885	0.5880	0.0004	0.1202	0.0739	0.0004	0.0004	0.4963	0.0133	0.5778	0.0805
PTS-PLGA vs. Cd	<0.000	<0.000	<0.000	<0.000	<0.000	<0.000	<0.000	<0.000	<0.000	<0.000	0.0003	<0.000
PTS-PLGA vs. Cd+PTS	<0.000	0.0020	0.0493	<0.000	0.0005	<0.000	<0.000	<0.000	0.0001	<0.000	0.0041	0.0006
PTS-PLGA vs. Cd+PTS-PLGA	0.1636	0.7553	0.5433	0.0003	0.0994	0.0240	0.0002	0.0002	0.3768	0.0054	0.2751	0.0581
Cd vs. Cd+PTS	0.0012	0.0303	0.0101	0.0070	0.0874	0.0031	0.8652	0.4163	0.0073	0.0127	0.5611	0.0006
Cd vs. Cd+PTS-PLGA	<0.000	<0.000	0.0007	<0.000	0.0004	<0.000	0.0046	<0.000	<0.000	<0.000	0.0106	<0.000
Cd+PTS vs. Cd+PTS-PLGA	0.0003	0.0186	0.5880	0.0026	0.0553	<0.000	0.0295	<0.000	0.0027	0.0021	0.1745	0.1296

Abbreviations: APV, adjusted p-value; PTS, pterostilbene; PTS-PLGA, pterostilbene-loaded poly(lactic-co-glycolic acid) nanoparticles; Cd, cadmium; KIM-1, kidney injury molecule-1; Nrf2, nuclear factor erythroid 2-related factor 2; HO-1, heme oxygenase-1; GSH, reduced glutathione; SOD, superoxide dismutase; CAT, catalase; GPx, glutathione peroxidase; mRNA, messenger RNA; ELISA, enzyme-linked immunosorbent assay.

Table S3. Adjusted *p*-values from Tukey's HSD multiple-comparison test for renal oxidative damage and inflammatory parameters following Cd exposure and treatment with free PTS or PTS-PLGA.

Groups / Assay	**ROS**	**MDA**	**PC**	**8-OHDG**	**TLR4 ELISA**	**TLR4 mRNA**	**NF-κB ELISA**	**NF-κB mRNA**	**COX-2 ELISA**	**COX-2 mRNA**	**TNF-α**	**IL-6**	**IL-1β**
Tukey's multiple comparisons test	APV	APV	APV	APV	APV	APV	APV	APV	APV	APV	APV	APV	APV
Control vs. PTS-PLGA	0.9568	0.8555	0.9953	0.9869	>0.9999	>0.9999	>0.9999	0.9968	0.9433	>0.9999	0.9994	0.9998	>0.9999
Control vs. PTS-PLGA	0.6471	0.7428	0.9755	0.9291	>0.9999	0.9999	0.9998	0.9882	0.7452	>0.9999	0.9985	0.9982	0.9995
Control vs. Cd	<0.000	<0.000	<0.000	<0.000	<0.000	<0.000	<0.000	<0.000	<0.000	<0.000	<0.000	<0.000	<0.000
Control vs. Cd+PTS	<0.000	<0.000	<0.000	0.0001	<0.000	<0.000	0.0650	0.0444	<0.000	<0.000	0.0001	<0.000	0.0061
Control vs. Cd+PTS-PLGA	0.0187	0.0031	0.0001	0.9554	0.3008	0.1151	0.9964	0.8197	<0.000	0.0012	0.5110	0.0693	0.9645
PTS-PLGA vs. PTS-PLGA	0.9763	0.9999	0.9999	0.9995	>0.9999	>0.9999	>0.9999	>0.9999	0.9960	>0.9999	>0.9999	>0.9999	>0.9999
PTS-PLGA vs. Cd	<0.000	<0.000	<0.000	<0.000	<0.000	<0.000	<0.000	<0.000	<0.000	<0.000	<0.000	<0.000	<0.000
PTS-PLGA vs. Cd+PTS	<0.000	<0.000	<0.000	<0.000	<0.000	<0.000	0.0483	0.0205	<0.000	<0.000	<0.000	<0.000	0.0045
PTS-PLGA vs. Cd+PTS-PLGA	0.0047	0.0005	<0.000	0.6951	0.2739	0.0889	0.9844	0.5693	<0.000	0.0011	0.3467	0.0450	0.9171
PTS-PLGA vs. Cd	<0.000	<0.000	<0.000	<0.000	<0.000	<0.000	<0.000	<0.000	<0.000	<0.000	<0.000	<0.000	<0.000
PTS-PLGA vs. Cd+PTS	<0.000	<0.000	<0.000	<0.000	<0.000	<0.000	0.0425	0.0159	<0.000	<0.000	<0.000	<0.000	0.0037
PTS-PLGA vs. Cd+PTS-PLGA	0.0015	0.0004	<0.000	0.5172	0.2469	0.0779	0.9748	0.4840	<0.000	0.0009	0.3163	0.0351	0.8749
Cd vs. Cd+PTS	<0.000	0.0593	<0.000	<0.000	0.1489	0.0001	0.0029	0.0027	0.1232	0.6261	0.5993	0.0080	0.0010
Cd vs. Cd+PTS-PLGA	<0.000	<0.000	<0.000	<0.000	<0.000	<0.000	<0.000	0.0001	<0.000	<0.000	0.0001	<0.000	<0.000
Cd+PTS vs. Cd+PTS-PLGA	<0.000	<0.000	0.0001	0.0004	0.0008	<0.000	0.1394	0.3005	<0.000	0.0002	0.0015	0.0091	0.0227

Abbreviations: APV, adjusted *p*-value; PTS, pterostilbene; PTS-PLGA, pterostilbene-loaded poly(lactic-co-glycolic acid) nanoparticles; Cd, cadmium; ROS, reactive oxygen species; MDA, malondialdehyde; PC, protein carbonyl; 8-OHdG, 8-hydroxy-2′-deoxyguanosine; TLR4, Toll-like receptor 4; NF-κB, nuclear factor kappa B; COX-2, cyclooxygenase-2; TNF-α, tumor necrosis factor-alpha; IL-6, interleukin-6; IL-1β, interleukin-1 beta; mRNA, messenger RNA; ELISA, enzyme-linked immunosorbent assay.

Table S4. Adjusted *p*-values from Tukey's HSD multiple-comparison test for renal apoptosis-related parameters, histopathological damage scores, and Nrf2 and NF-κB immunoreactivity following Cd exposure and treatment with free PTS or PTS-PLGA.

**Groups** / **Assay**	**Cas-3**	**Cas-8**	**Bax/Bcl2**	**P53**	**Damage Score**	**Nrf2 %**	**NF-κB**
Tukey's multiple comparisons test	APV	APV	APV	APV	APV	APV	APV
Control vs. PTS-PLGA	0.9996	0.9998	0.9997	>0.9999	>0.9999	0.9956	>0.9999
Control vs. PTS-PLGA	0.9872	0.9993	0.9908	0.9951	>0.9999	0.9988	>0.9999
Control vs. Cd	<0.0001	<0.0001	<0.0001	<0.0001	<0.0001	<0.0001	<0.0001
Control vs. Cd+PTS	0.0077	<0.0001	<0.0001	<0.0001	0.0195	0.0002	0.0034
Control vs. Cd+PTS-PLGA	0.9369	0.0173	0.0060	0.0038	0.5743	0.6369	0.0400
PTS-PLGA vs. PTS-PLGA	0.9994	>0.9999	0.9997	0.9974	>0.9999	0.9476	>0.9999
PTS-PLGA vs. Cd	<0.0001	<0.0001	<0.0001	<0.0001	<0.0001	<0.0001	<0.0001
PTS-PLGA vs. Cd+PTS	0.0047	<0.0001	<0.0001	<0.0001	0.0195	0.0004	0.0027
PTS-PLGA vs. Cd+PTS-PLGA	0.8262	0.0112	0.0038	0.0035	0.5743	0.8847	0.0312
PTS-PLGA vs. Cd	<0.0001	<0.0001	<0.0001	<0.0001	<0.0001	<0.0001	<0.0001
PTS-PLGA vs. Cd+PTS	0.0028	<0.0001	<0.0001	<0.0001	0.0195	0.0001	0.0028
PTS-PLGA vs. Cd+PTS-PLGA	0.6539	0.0098	0.0024	0.0017	0.5743	0.4281	0.0325
Cd vs. Cd+PTS	0.0030	0.0292	0.0005	0.0120	<0.0001	0.0002	<0.0001
Cd vs. Cd+PTS-PLGA	<0.0001	<0.0001	<0.0001	<0.0001	<0.0001	<0.0001	<0.0001
Cd+PTS vs. Cd+PTS-PLGA	0.0355	0.0245	0.0004	0.0002	0.5743	0.0019	0.6848

Abbreviations: APV, adjusted *p*-value; PTS, pterostilbene; PTS-PLGA, pterostilbene-loaded poly(lactic-co-glycolic acid) nanoparticles; Cd, cadmium; Cas-3, caspase-3; Cas-8, caspase-8; Bax, Bcl-2-associated X protein; Bcl-2, B-cell lymphoma 2; p53, tumor protein p53; Nrf2, nuclear factor erythroid 2-related factor 2; NF-κB, nuclear factor kappa B.
